# Optimizing lipopeptide bioactivity: The impact of non-ionic surfactant dressing

**DOI:** 10.1016/j.jpha.2024.101020

**Published:** 2024-06-08

**Authors:** Ágnes Ábrahám, Gergő Gyulai, Judith Mihály, Andrea Horváth, Orsolya Dobay, Zoltán Varga, Éva Kiss, Kata Horváti

**Affiliations:** a MTA-HUN-REN TTK Lendület “Momentum” Peptide-Based Vaccines Research Group, Institute of Materials and Environmental Chemistry, Research Centre for Natural Sciences, Budapest, H-1117, Hungary; b Laboratory of Interfaces and Nanostructures, Institute of Chemistry, Eötvös Loránd University, Budapest, H-1117, Hungary; c HUN-REN TTK Biological Nanochemistry Research Group, Institute of Materials and Environmental Chemistry, Research Centre for Natural Sciences, Budapest, H-1117, Hungary; d Institute of Medical Microbiology, Semmelweis University, Budapest, H-1085, Hungary; e Department of Physical Chemistry and Materials Science, Faculty of Chemical Technology and Biotechnology, Budapest University of Technology and Economics, Budapest, H-1111, Hungary

**Keywords:** Lipopeptide, Pluronic, Poloxamer, Multi-epitope vaccine, Antimicrobial peptide

## Abstract

The aim of the research is to increase the applicability of lipopeptides as drugs. To this end, non-ionic triblock copolymers, namely poloxamers, were applied. The physico-chemical properties of poloxamers vary depending on the length of the blocks. In our study, we experimented with different types and systematically investigated the variation of the critical micelle concentration (CMC) of poloxamers at 25 and 37 °C in different media. In addition, the cytotoxicity of the different poloxamer micelles on three different cell lines was evaluated, and based on the results, Plur104, Plur123, and Plur127 were selected. Fatty acid elongated derivatives of a short antibacterial peptide (pL1), a medium-sized anticancer peptide (pCM15), and a branched-chain vaccine antigen (pATIPC) were used as lipopeptide models, and their formulations with the selected poloxamers were investigated. The solubility and homogeneity of the lipopeptides were significantly increased, and dynamic light scattering (DLS) measurements showed the formation of small particles of around 20 nm, which were well reproducible and storable. Similar homogenous micelle formation was observed after freeze-drying and reconstitution with water. The pL1 lipopeptide, formulated with the selected poloxamers, exhibited enhanced antibacterial activity with significantly reduced haemolytic side effects. The pCM15 peptide, when incorporated into poloxamer micelles, showed significantly enhanced cytotoxicity against tumor cells. Additionally, the internalization rate of poloxamer-formulated pATIPC peptide by antigen-presenting model cells exceeded that of the unformulated peptide. Our results demonstrate the potential of poloxamers as promising tools for the formulation of lipopeptides and for the optimization of their selectivity.

## Highlights

• Poloxamer formulation improves the applicability of lipopeptides.
• Antibacterial efficacy was boosted, while the haemolytic activity was minimized.
• Freeze-dried form of poloxamer–lipopeptide systems is stable and storable.
• Peptide chain length is a key determinant of the mixed micelle characteristics.
• Experimental CMC values of poloxamers in relevant biological conditions are reported.

## Graphical abstract

**Figure:**
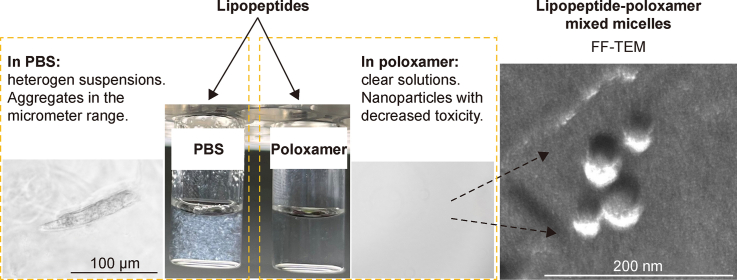

## 1 Introduction

Lipopeptides have gained significant attention as a potential new class of drug molecules. They have been extensively studied for their antimicrobial [1] and anticancer activity [2], for controlling blood sugar level and obesity [3], but also for their biopesticide activity, as lipopeptides can inhibit the growth of several phytopathogens [4,5]. Besides, lipopeptide constructs have been explored in vaccine development as promising candidates for safer, fully synthetic self-adjuvanting antigens [6,7]. There are 14 lipopeptides in the clinics so far, of which five drugs (semaglutide, tirzepatide, dalbavancin, insulin degludec, and rezafungin) received approval in the last decade (Table S1) [[8], [9]].

Nevertheless, lipopeptides generally violate the medicinal chemistry filters, such as the Lipinski's rule of five, Veber filter, and Ghose filter [10], mainly due to their molecular weight (MW) and log*P* characteristics. Hydrophobicity is the main drawback of using lipopeptides, not only due to the low water solubility, but also because of the heterogeneity of their suspensions. The most convenient sterilization method is membrane filtration, wherein the product is passed through a 0.22 μm pore size filter and filled into pre-sterilized containers in an aseptic processing environment. Lipopeptides generally form aggregates in the micrometer range, upon simple water or medium solvation; therefore, filtration is not a suitable method to ensure product sterility. Additionally, low aqueous solubility poses a challenge in conducting cell-based assays and often leads to failure during drug development. Lipopeptides typically exhibit high protein binding affinity, and they can also interact with the protein components of cell culture media, resulting in additional insolubility and precipitation.

The use of organic solvents, such as dimethyl sulfoxide (DMSO), for the pre-solvation of water-insoluble compounds, is a common method, but these organic solvents can impact the compound stability and protein/receptor binding, and may lead to undesired effects on the cell cultures [11]. Due to its cytotoxicity, the percentage of DMSO in the culture media must be kept below 0.1%–1% (*V/V*), which is typically insufficient for the proper solubilization of lipopeptides.

The addition of common hydrotropic agents such as sodium citrate, sodium acetate, sodium salicylate, nicotinamide, etc., which are included in the pharmacopoeia of several countries, is usually insufficient for the successf solubilization of lipopeptide drug candidates.However, the use of nanomedicine-based drug-delivery and encapsulation techniques can help to overcome these obstacles. Micelles are attractive delivery systems due to their small particle size, which allows for sterile filtration but also enhances penetration to target tissues. The incorporation of lipopeptide molecules into the hydrophobic core of typical micelle formulations can be very efficient, owing to hydrophobic-hydrophobic interactions.

In this study, self-assembling triblock copolymers, namely poloxamers, were investigated to control the size of aggregates and *in vitro* properties of biologically active lipopeptides. For model compounds, three different types of lipopeptides were chosen: 1) a short cationic peptide with fatty acid elongation, bearing antibacterial efficacy, 2) a medium-sized lipopeptide with anticancer activity, and 3) a branched -chain multiepitope construct consisting of 38 amino acids. The molecules differ in terms of size, hydrophobicity, and structural characteristics, making them suitable for obtaining an overall picture of the effect of poloxamer dressing of lipopeptides.

Poloxamers are non-ionic amphiphilic copolymers consisting of poly(ethylene oxide) (PEO)-poly(propylene oxide) (PPO)-PEO blocks. Their versatility arises from their tunable properties, which are linked to the length of their hydrophilic and hydrophobic chains. They are applied in various fields, serving as surfactants, solubilizers, wetting agents, emulsifiers, and adsorption enhancers. Due to their biocompatibility, they are also promising materials in medical applications, both as matrices and carriers [12,13], or surface modifiers [[14], [15], [16], [17]]. Therefore, the U.S. Food and Drug Administration (US FDA) and European Medicines Agency (EMA) have approved numerous poloxamers for human use.

**Fig. 1:**
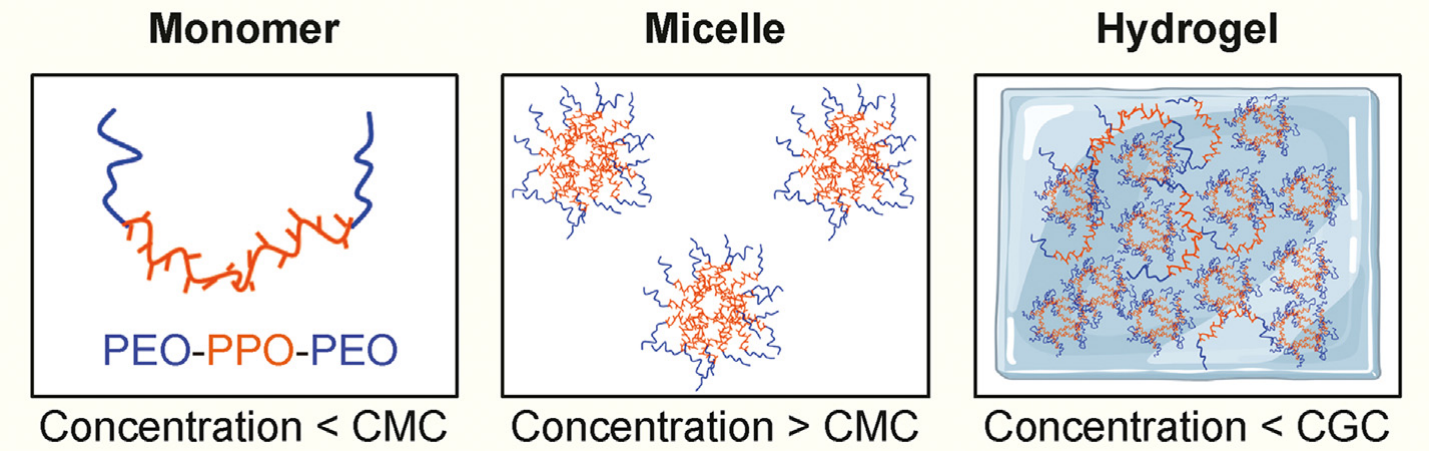
Schematic illustration of the solution structure with increasing poloxamer concentration. PEO: poly(ethylene oxide); PPO: poly(propylene oxide); CMC: critical micelle concentration; CGC: critical gelation concentration. Figure was created with BioRender.com.

Poloxamers can encapsulate pharmaceutical agents within the core, constructed by the PPO segment, and within the region of the PEO-based corona [13,18]. The hydrophilic PEO region acts as a protective shield, prolonging drug circulation time within the body, effectively extending the half-life and enhancing steric stability. Poloxamer copolymers can form either direct or reverse micelles depending on the characteristics of the external solvent at the critical micelle concentration (CMC) (Fig. 1). These micelles have small size, typically ranging from 10 to 100 nm, and exhibit spherical or rod-like shapes [12]. By modulating the hydrophilic and hydrophobic segments, the size of the hydrophobic core and the colloidal properties can be optimized. Polymer micelles, which are more resistant to dilution than conventional surfactants, can be produced on a large scale [19].

Poloxamer copolymers have been investigated as potential carriers for peptides and lipopeptides. The main focus of these studies has been on systems with poloxamer concentrations above their CMC, resulting in the creation of hydrogels. Additionally, poloxamers have been utilized in various vaccine formulations since copolymers with high hydrophobicity have been found to exhibit strong adjuvant activity [20,21]. Various types of poloxamer compounds have been utilized as a prolonged drug release vehicle and as a stabilizing agent for both the antigen and adjuvant components present in the matrix [22].

The goal was to investigate a wide range of available poloxamers. However, it was decided to exclude those with the highest hydrophobicity during the selection process because they are less likely to form micelles. To aid in the selection process, the hydrophilic-lipophilic balance (HLB) values were used. The HLB is a parameter that characterizes the amphiphilic nature of the surfactant and is closely related to the type and size of micelles, encapsulation capacity, stability, and drug release profile. The poloxamers chosen for this study exhibit diversity, varying in factors such as the number of PEO and PPO monomers, and MW. Table 1 presents the characteristics of the polymers used, while Table S2 [[13], [23], [24], [25], [26], [27], [28], [29]] presents the CMC data found in the literature.

**Table 1:** Poloxamers used in this study, together with their trade names and characteristics.

Poloxamer name	Pluronic name	Abbreviation in this study	MW	EO units	PO units	HLB values	Distributor
184	L-64	Plur64	2,900	13	30	15	BASF Hungária
188	F-68	Plur68	8,400	76	30	29	BASF Hungária
234	P-84	Plur84	4,200	19	39	14	BASF Hungária
333	P-103	Plur103	4,950	17	56	9	BASF Hungária
334	P-104	Plur104	5,900	27	56	13	BASF Hungária
335	P-105	Plur105	6,500	37	56	15	BASF Hungária
403	P-123	Plur123	5,800	20	70	8	Sigma-Aldrich
407	F-127	Plur127	12,600	97	63	22	Sigma-Aldrich

The state of the poloxamer is marked with liquid (L), flakes (F), and paste (P). MW: molecular weight; EO: ethylene oxide; PO: propylene oxide; HLB: hydrophilic-lipophilic balance.

## 2 Materials and methods

### 2.1 Materials

Various PEO-PPO-PEO triblock copolymers were used to prepare peptide-loaded poloxamer micelles. The properties of these copolymers are collected in Table 1. They were applied as received. The composition of the polymers is characterized by the average length of the PPO and the PEO chains, which is also shown in Table 1.

Pyrene (C_16_H_10_), which is a fluorescent dye, was provided by Merck (Budapest, Hungary) and was used for the CMC determination.

For the peptide synthesis, amino acid derivatives, resins, and OxymaPure coupling reagent were obtained from Iris Biotech (Darmstadt, Germany). Reagents such as *N,N′*-diisopropylcarbodiimide (DIC), triisopropylsilane (TIS), 1-hydroxybenzotriazole (HOBt), 1,8-diazabicyclo[5.4.0]undec-7-ene (DBU), and 5(6)-carboxyfluorescein (Cf) were purchased from Merck. Trifluoroacetic acid (TFA) and acetonitrile were liquid chromatography-mass spectrometry (LC-MS) grade and purchased from VWR International Kft. (Budapest, Hungary). *N,N*-dimethylformamide (DMF), dichloromethane (DCM), dimethyl sulfoxide (DMSO), and diethyl ether were puriss grade and purchased from Novochem (Budapest, Hungary).

For the *in vitro* assays, Roswell Park Memorial Institute (RPMI)-1640 medium, Dulbecco's Modified Eagle Medium (DMEM), fetal bovine serum (FBS), 2 mM l-glutamine, trypsin, non-essential amino acids, and penicillin-streptomycin were from Thermo Fisher Scientific Inc. (Gibco; Waltham, MA, USA). HPMI (HEPES-modified RPMI) buffer (9 mM glucose, 10 mM NaHCO_3_, 119 mM NaCl, 9 mM 4-(2-hydroxyethyl)piperazine-1-ethane-sulfonic acid (HEPES), 5 mM KCl, 0.85 mM MgCl_2_, 0.053 mM CaCl_2_, and 5 mM Na_2_HPO_4_ ‧ 2H_2_O; pH 7.4) was prepared in-house using components obtained from Merck. Gibco phosphate-buffered saline (PBS; pH 7.4) was purchased from Life Technologies Magyarország Kft (Budapest, Hungary).

### 2.2 Determination of the CMC of the selected poloxamers

The CMC of the poloxamers in different media was determined using the fluorescence method with pyrene as the fluorescence probe [24,30]. The media for the poloxamer solutions were water, PBS, and PBS with 5% DMSO content.

For the experiments, 0.5 mL of a 1.2 mM pyrene solution in acetone was added to 1 L of the chosen medium, left stirring overnight, and then filtered. A series of poloxamer solutions with increasing concentrations were prepared using this filtered pyrene solution. After allowing the samples to equilibrate for 2 h, measurements were conducted with a Varian Cary Eclipse fluorescence spectrophotometer (Varian Inc., Santa Clara, CA, USA) (right-angle geometry, 1 cm × 1 cm quartz cell) at 25.00 ± 0.02 and 37.00 ± 0.02 °C. The excitation wavelength was set at 320 nm with a monochromator slit width of 5 nm, while the emission slit was set to 2.5 nm. Due to the sensitivity of the pyrene to the polarity of the medium, different spectral patterns can be observed in micellar and non-micellar solutions. Based on this, the ratio of the intensity of the first peak (*I*_1_; at 373 nm) to the third peak (*I*_3_; at 383 nm) is a sensitive parameter characterizing the polarity of the probe's environment. Low *I*_1_/*I*_3_ values indicate an environment with low polarity, which can be used as a sign of micelle formation, reflecting the solubilization of pyrene into a less polar environment [31]. For determining the CMC, the ln(concentration) plotted against *I*_1_/*I*_3_ graphs were analyzed.

### 2.3 Peptide synthesis, purification, and analytical characterization

Peptide L1 was synthesized on Fmoc-Rink Amide MBHA resin (capacity = 0.67 mmol/g) in an automated peptide synthesizer (Syro-I; Biotage, Uppsala, Sweden) using standard Fmoc/*t*Bu strategy with DIC/HOBt coupling reagents. The synthetically more challenging CM15 peptide and the linear backbone of ATIPC peptide were synthesized at the Institute of Chemistry, Eötvös Loránd University (Budapest, Hungary) using flow peptide synthesis [32,33]. To follow the internalization of the vaccine conjugate, pATIPC was also synthesized in a Cf-labelled version. The Cf-moiety was introduced on the *N*-terminus of the branched peptide.

Reverse phase high-performance liquid chromatography (RP-HPLC) purification was performed on an UltiMate 3000 Semiprep HPLC (Thermo Fisher Scientific Inc.) with a Phenomenex Jupiter Proteo C_12_ column (250 mm × 10 mm, 4 μm) using gradient elution, consisting of 0.1% TFA in water (eluent A) and 0.1% TFA in acetonitrile/water (4:1, *V/V*) mixture (eluent B). The purity of the peptides was analyzed on a Shimadzu RP-HPLC system using a Phenomenex Jupiter Proteo C_12_ column (150 mm × 4.6 mm, 4 μm). The flow rate was 1 mL/min, and the absorbance was detected at λ = 220 nm. The high-resolution mass spectrum of the purified peptides was measured on a Thermo Scientific Q Exactive Focus Hybrid Quadrupole-Orbitrap Mass Spectrometer (Thermo Fisher Scientific Inc.).

A detailed description of the synthesis and the results of the mass spectrometric analysis can be found in Section S1 and Figs. S1–S3 in the Supplementary data.

### 2.4 Preparation and characterization of the peptide-loaded poloxamer micelles

Peptide-loaded poloxamer micelles were prepared by solubilizing the peptides in 50 g/L poloxamer solutions in PBS (pH 7.4). The peptides were previously dissolved in DMSO, and then the poloxamer solutions were added. The final DMSO content was 5%. The solutions were left shaking overnight to allow enough time for solubilization. Before the measurements, PBS was added to the solutions to reach 5 g/L poloxamer concentration. In the biological experiments, these samples were diluted twice with the corresponding culture media.

The average hydrodynamic diameter (*d*_H_) and polydispersity of the peptide-loaded micelles were determined using a dynamic light scattering (DLS) instrument (NanoLab 3D^TM^, LS Instruments, Fribourg, Switzerland) based on three-dimensional (3D) modulation technology. A fibre-coupled diode laser operating at 638 nm served as the light source. The measurements were performed at a detection angle of 90° and at temperatures of 37.00 ± 0.02 °C. The recorded autocorrelation functions were analyzed using the second order cumulant expansion method. Before the measurements, the samples were filtered with a 0.2 μm syringe filter.

Size and morphology of pL1-Plu123 mixed micelles were also studied by freeze-fracturetransmission electron microscopy (FF-TEM). First, 30 μL of the previously prepared micelles, containing 800 μM pL1 peptide and 20 g/L Plur123 in H_2_O, were added to 12.4 μL glycerin and approximately 2 μL was transferred onto a gold sample holder. After the sample was rapidly frozen in liquid freon, fracturing was performed in a Blazers BAF 400D (Blazers, Lichtenstein) FF device. The replica of the fractured surface was prepared with platinum-carbon evaporation, followed by cleaning with distilled H_2_O, and transferred to a 200-mesh copper grid for imaging in a Morgagni 268D transmission electron microscope (Field Electron and Ion Company, FEI, Eindhoven, the Netherlands).

### 2.5 Infrared (IR) spectroscopy

To study peptide-poloxamer interaction, the pL1 and Plur123 samples were selected and inspected by IR spectroscopy using a Varian 2000 (Scimitar Series) FT-IR spectrometer (Varian Inc., Palo Alto, CA, USA). The liquid samples were measured using the attenuated total reflectance (ATR) technique (‘Golden Gate’ single reflection diamond ATR, Specac Ltd., Orpington, UK): 3 μL of sample was dropped on the diamond ATR unit and dried by a gentle N_2_ stream. All spectra were collected at a spectral resolution of 2 cm^−1^ with 64 scans. Spectral manipulations (ATR correction, spectral subtraction) were performed using the GRAMS/AI (7.02) software (Galactic Inc., Milwaukee, WI, USA).

### 2.6 Bacteria, human cells, and culture conditions

Methicillin-susceptible *Staphylococcus aureus* (*S. aureus*) (MSSA) (ATCC No. 29213) and methicillin-resistant *S*. *aureus* (MRSA) (ATCC No. 33591) were grown in Mueller Hinton Broth (Biolab Zrt. Budapest, Hungary).

HT-29, human colorectal adenocarcinoma cell line (ECACC No. 91072201; Merck), MonoMac-6 human monocytic cell line (DSMZ No. ACC 124; Braunschweig, Germany), and HepG2 human hepatoblastoma cell line (ATCC No. HB-8065; Merck) were maintained as an adherent culture in RPMI-1640 medium, supplemented with 10% heat-inactivated FBS, l-glutamine (2 mM), and penicillin-streptomycin (100−100 units/mL) at 37 °C in a humidified atmosphere containing 5% CO_2_. VERO-E6 cells (non-human primate origin *Cercopithecus aethiops*, kidney; ECACC No. 85020206; Merck) were maintained in DMEM high-glucose (4.5 g/L) medium containing 10% FBS and supplemented with 2 mM of l-glutamine and 1 mM sodium pyruvate. For the haemolysis assay, concentrate of human red blood cells (RBCs) was obtained from healthy volunteers and the Hungarian National Blood Transfusion Service (Budapest, Hungary).

### 2.7 Determination of cytotoxicity and haemolytic activity of the poloxamers

Viability of human model cells, namely MonoMac-6 monocytes, HepG2 hepatoma cells, and VERO-E6 kidney cells, after treatment with the poloxamer compounds was tested using the AlamarBlue viability assay. Cells were seeded one day prior to the experiment in a 96-well tissue culture plate (Sarstedt, Nümbrecht, Germany) at a density of 10,000 cells in 100 μL medium per well. In the following day, cells were treated with a three-fold serial dilution of the compounds. The highest treatment concentrations were calculated based on the CMC values found in the literature (Table S2). After 24 h of incubation at 37 °C in 5% CO_2_, cells were washed three times, then 10% (*V/V*) AlamarBlue reagent solution (resazurin sodium salt (Merck), dissolved in PBS at 0.15 mg/mL, pH 7.4) was added to each well. Following 3 h of incubation, the fluorescence was detected (λ_Ex_ = 530/30 nm and λ_Em_ = 610/10 nm) using a Synergy H4 multimode microplate reader (BioTek, Winooski, VT, USA). All measurements were performed in quadruplicate, and the viability of the compound-treated cells was compared to untreated control cells. All data are presented as mean ± standard deviation (SD), and the half-maximal inhibitory concentration (IC_50_) values were calculated from the dose-response curves after fitting with non-linear regression. Microscopic images of the cells were also captured using an Olympus CX41 microscope (objective 40×) (Olympus Europa, Hamburg, Germany). Haemolytic activity of the compounds was tested on human RBC. First, RBCs were washed twice with PBS, then diluted with PBS (2% (*V/V*) RBC) and plated on a 96-well, round-bottom plate at a volume of 50 μL. Compounds were prepared at a volume of 50 μL and added to the erythrocytes. The final RBC concentrate was 1% (*V/V*), and the final volume was 100 μL in the wells. Plates were incubated at 37 °C, 5% CO_2_ for 2 h, then centrifuged (2,000 rpm, 5 min, 4 °C). Then, 50 μL of the supernatants were carefully transferred to a new plate, containing 100 μL H_2_O in each well. Optical density (OD) was measured at 414 and 450 nm by a Synergy H4 multimode microplate reader and the percentage of haemolysis was compared to a positive control, namely the bee venom melittin (10 μM, in PBS). All data presented as the mean ± SD (*n =* 4).

### 2.8 Antibacterial effect

The antibacterial activity of pL1 peptide and its poloxamer derivatives was determined using the broth microdilution method [34]. Fresh overnight cultures of MSSA and MRSA were used to prepare 0.5 McFarland suspensions in 0.9% NaCl. This was further diluted 1:100 in Mueller-Hinton (MH) broth, and 50 μL was aliquoted to each well on a 96-well round bottom plate (Sarstedt). Growth control (in the absence of the antimicrobial compounds) and sterile control (no bacteria and no drug) were also applied in eight parallels on each microtiter plate.

pL1 peptide was dissolved at a concentration of 40 mM in DMSO, and 4.5 μL of this solution was added to 85.5 μL PBS or poloxamer solution (50 g/L in PBS), then diluted 10 times with MH broth immediately before the experiment. Ten-step, two-fold dilution series of the compounds were prepared in MH broth, and 50 μL of each dilution was added to the bacterial suspension. The final concentration of bacteria was 5 × 10^5^ CFU/mL. Plates were incubated in ambient air for 18−20 h at 35 °C. Minimal inhibitory concentration (MIC) was defined as the lowest concentration of a compound at which no visible growth of the bacteria occurred, as determined by the naked eye. In addition, OD was also determined at 595 nm using a Multiscan FC plate reader (Thermo Fisher Scientific Inc.). All measurements were performed in duplicates, and the mean bacterial density relative to the untreated control wells was graphically presented, together with the SD.

### 2.9 *In vitro* antitumor effect

The antitumor activity of pCM15, with or without poloxamer formulation, was investigated on HT-29 colorectal adenocarcinoma cells. Cells were seeded one day prior to the experiment, then treated with 0.2–50 μM pCM15 peptide, either unformulated or formulated with poloxamers. After 24 h of incubation at 37 °C in 5% CO_2_, cells were washed three times, and then a 10% (*V/V*) AlamarBlue reagent solution (Merck) was added to each well. Following 3 h of incubation, the fluorescence was detected (λ_ex_ = 530 and λ_em_ = 610), and the cytotoxicity of the compounds was determined, compared to untreated control cells. All data are presented as mean ± SD (*n =* 4). Statistical analysis was performed using one-way analysis of variance (ANOVA), followed by Tukey's post-hoc test. Statistical significances were denoted as ^∗^*P <* 0.05, ^∗∗^*P <* 0.01, and ^∗∗∗^*P* < 0.001.

### 2.10 Internalization study

Internalization of the Cf-labelled pATIPC peptide, with or without poloxamer dressing, was measured in MonoMac-6 human monocytes. One day before the experiment, cells were seeded in a 24-well tissue culture plate (100,000 cells/well in 1 mL medium). The next day, cells were treated with 2.5 μM peptide, either with or without poloxamers. After 2 h of incubation, cells were washed with serum-free RPMI medium. Then, the supernatant was removed, and 100 μL of 0.25% trypsin was added to the wells. After 5 min of incubation, 0.8 mL of 10% FBS/HPMI medium was added. Subsequently, cells were washed and re-suspended in 0.3 mL HPMI medium. The intracellular fluorescence intensity of the cells was measured on a BD LSR II flow cytometer (BD Biosciences, Franklin Lakes, NJ, USA) on channel fluorescein isothiocyanate (FITC) (emission at λ = 505 nm), and the data was analyzed with a FACSDiva 5.0 software (BD Biosciences). All measurements were performed in triplicates, and the mean fluorescent intensity together with SD was graphically presented. Statistical analysis was performed using one-way ANOVA, followed by Tukey's post-hoc test. Statistical significances were denoted as ^∗^*P <* 0.05, ^∗∗^*P <* 0.01, and ^∗∗∗^*P* < 0.001.

## 3 Results and discussion

A panel of eight poloxamers was selected to cover a wide range both in terms of polymer size and number of ethylene oxide (EO) and propylene oxide (PO) monomer units (Table 1). The panel includes poloxamers found in the European and US pharmacopoeia, namely poloxamer 188 and 407 (referred to as Plur68 and Plur127, respectively). However, other pharmacopoeial types, such as poloxamer 124, 237, and 338, were excluded from the polymers tested. The study aimed to comprehensively understand the potential applications of poloxamers in the formation of mixed micelles with lipopeptides by determining the extent to which polymers with varying HLB properties can be used. Detailed characterization of the poloxamers is shown in Section S2 and Fig. S4 in the Supplementary data.

### 3.1 Cytotoxic effect of the selected poloxamers

As a primary step, the cytotoxicity of the poloxamers used for lipopeptide encapsulation was thoroughly investigated to ensure their biocompatibility for the planed cell-based assays. Various concentrations of the polymers were employed for the measurements, a comprehensive assessment of their safety profile and selection of the most suitable poloxamers for the peptide encapsulation process. The highest concentration was selected based on the CMC values (Table S2) [[13], [23], [24], [25], [26], [27], [28], [29]] reported in the literature as a guide.

The effect of poloxamers on human cells was investigated using a colorimetric viability assay. Three different cell lines were used for this study: 1) the human MonoMac-6 cell line, which exhibits stable phenotypic and functional characteristics of mature blood monocytes and is therefore useful model for *in vitro* studies of monocyte biology and toxicity towards blood cells [35]. 2) VERO-E6, African green monkey kidney cells, were utilized as a suitable model to test the *in vitro* nephrotoxic potential of drugs [36]. Besides, VERO-E6 cells are broadly applied as hosts for antiviral assays and vaccine production, and are widely used to isolate, propagate and study different viruses such as severe acute respiratory syndrome coronavirus 2 (SARS-CoV-2), poliovirus, influenza virus, dengue virus, etc. [37,38]. 3) Lastly, the HepG2 human hepatoma cell line served as a hepatotoxicity cell model, frequently used in predicting liver toxicity and metabolism of drug compounds [39].

The results of the viability assays (Fig. S5) and the calculated IC_50_ values (Table 2) indicated that Plur68, Plur104, Plur123, and Plur127 were not toxic to the cells even at the highest tested concentration. Therefore, they can be applied for the formulation of lipopeptide-based bioactive compounds.

**Table 2:** The half-maximal inhibitory concentration (IC_50_) values of the poloxamers, measured on different human cell lines.

Poloxamer	MonoMac-6 IC_50_ ± SD (μM)	VERO-E6 IC_50_ ± SD (μM)	HepG2 IC_50_ ± SD (μM)
Plur64	214.8 ± 25.6	232.5 ± 14.2	53.0 ± 7.1
Plur68	>850	>850.0	>850.0
Plur84	544.0 ± 15.9	245.3 ± 25.4	14.5 ± 1.8
Plur103	151.3 ± 19.6	>500.0	25.8 ± 6.2
Plur104	>500.0	>500.0	462.8 ± 25.3
Plur105	137.5 ± 16.8	173.4 ± 21.0	22.0 ± 2.4
Plur123	>500.0	>500.0	>500.0
Plur127	>500.0	>500.0	>500.0

The IC_50_ values were defined as the poloxamer concentration that caused 50% cell death, obtained from the dose-response curves, after fitting with non-linear regression. Data were presented as mean IC_50_ values ± standard deviation (SD) (*n =* 4).

In a recent study [40], *in vitro* cytotoxicity of Plur68 and Plur127 against HepG2, Caco-2, and MCF-7 was measured, and the results were in line with our observations. Namely, the poloxamer micelles were not cytotoxic with respect to all cell lines employed. In case of Plur123, Liu et al. [41] studied the cytotoxicity on HepG2, MCF-7, and B16 cell lines and found no effect on the cell viability at the concentration applied.

In addition to the cell viability tests, to study the effect of poloxamers on cell morphology, microscopic images were also captured (Fig. S6). Microscopic images showed serious cell membrane disruption and cell death in the case of treatment with Plur64, Plur84, Plur103, and Plur105, at concentration between 2 and 8 g/L. Generally, poloxamers have relatively low cytotoxicity or even a cytoprotective effect. However, some of the polymers exhibited significant toxicity towards mammalian cells [42]. Different mechanisms have been proposed to explain membrane disruption or interaction of poloxamers with cell membranes, in which the insertion of the hydrophobic part and absorption of the hydrophilic part seems to be the key elements [43,44]. The correlation of the HLB values with membrane permeabilization property has also been proposed, and in most of the cases, the relationship is clear. For example, the relatively hydrophilic Plur68 (HLB of 29) tends to absorb on the cell surface and acts as a cytoprotectant compound by contributing to the maintenance of the integrity of damaged cell membranes [[45], [46], [47]]. In contrast, the insertion of the more hydrophobic Plur64 (HLB of 15) into the lipid membranes was more dominant and led to membrane disruption. Houang et al. [48], in a study on the interaction of poloxamers with lipid bilayers using all-atom molecular dynamics, found that the ratio of PEO to PPO was the most important factor in determining the membrane insertion mechanism. Our results were in line with these observations, especially when cell membrane damage was detected in the case of Plur64 (HLB of 15), Plur84 (HLB of 14), Plur103 (HLB of 9), and Plur105 (HLB of 15). However, the intact cell membrane observed for the remaining polymers was not as obvious, considering the higher HLB values in the case of Plur68 (HLB of 29) and Plur127 (HLB of 22), and the lower HLB value in the case of Plur123 (HLB of 8). Therefore, it can be concluded that besides HLB values, other property indicators need to be considered. Overall, based on the comparative studies conducted, Plur68, Plur104, Plur123, and Plur127 have exhibited promising characteristics and appear to be highly suitable candidates for the peptide encapsulation process. However, due to its very high CMC value, Plur68 has been excluded from further experiments.

### 3.2 CMC of the selected poloxamers

When encapsulating a lipopeptide into poloxamer micelles, it is important to consider the concentration used. It is worth noting that the CMC values reported in the literature can vary widely for the same poloxamer and have mostly been determined in aqueous environments [23,24,[26], [27], [28], [29]]. However, in the current study, systems containing PBS buffer with DMSO are being investigated, which may influence the CMC value. To address this aspect, the CMCs of poloxamers in different media at 25 and 37 °C were experimentally determined (Table 3) using a pyrene fluorescence probe method [24,30,31]. Experimental details are shown in Section S2 and Fig. S4 in the Supplementary data.

**Table 3:** Critical micelle concentration (CMC) values of the poloxamers, determined by the fluorescence probe method at different temperatures and in different media.

Poloxamer	Medium	Temperature (°C)	CMC ± SD (g/L)
Plur104	Water	25	0.155 ± 0.008
	PBS	25	0.043 ± 0.003
	Water with 5% DMSO	25	0.073 ± 0.004
	PBS with 5% DMSO	25	0.040 ± 0.003
	Water	37	0.021 ± 0.001
	PBS	37	0.022 ± 0.001
	Water with 5% DMSO	37	0.069 ± 0.004
	PBS with 5% DMSO	37	0.047 ± 0.002
Plur123	Water	25	0.078 ± 0.006
	PBS	25	0.030 ± 0.002
	Water with 5% DMSO	25	0.067 ± 0.004
	PBS with 5% DMSO	25	0.033 ± 0.002
	Water	37	0.022 ± 0.001
	PBS	37	0.020 ± 0.001
	Water with 5% DMSO	37	0.042 ± 0.002
	PBS with 5% DMSO	37	0.040 ± 0.002
Plur127	Water	25	4.800 ± 0.190
	PBS	25	0.854 ± 0.060
	Water with 5% DMSO	25	2.431 ± 0.180
	PBS with 5% DMSO	25	0.505 ± 0.035
	Water	37	0.083 ± 0.003
	PBS	37	0.076 ± 0.004
	Water with 5% DMSO	37	0.183 ± 0.008
	PBS with 5% DMSO	37	0.124 ± 0.005

SD: standard deviation; PBS: phosphate-buffered saline; DMSO: dimethyl sulfoxide.

From the data, it can be observed that the CMC is affected by the presence of salt and DMSO at both temperatures. At 25 °C, the CMC is decreased by the presence of either substance in the system. The reduction is comparatively smaller when only DMSO is present, while the smallest CMC is observed when both salt and DMSO are present. For Plur104 and Plur123, the CMC decreases to approximately one-third, while for Plur127, it decreases to one-tenth. It has been observed that Plur127 is comparatively more hydrophilic and has a larger MW than Plur104 and Plur123. Additionally, the CMC is significantly reduced by the presence of electrolytes, which is consistent with previous research [[49], [50], [51]].

At 37 °C, the CMC values in PBS are comparable to or slightly lower than those in water. If DMSO is added to the solution, the CMC increases, approximately doubling or tripling. The most significant change in CMC is observed in the water-DMSO system. When both PBS and DMSO are present, the CMC value is slightly lower than when only DMSO is used. At 37 °C, the presence of salt reduces the CMC-increasing effect of DMSO, but even so, the CMC-increasing effect is still dominant. At 25 °C, it appears that the CMC may decrease in the presence of DMSO. However, at higher temperatures, it seems that the opposite effect may occur.

It is worthwhile to examine the changes that occur in the system as the temperature rises. Higher temperatures have been observed to reduce the hydration of the PEO block. Additionally, certain polar organic solvents have been found to reduce cohesive forces and increase the solubility of poloxamer molecules, resulting in an increased CMC [52]. In our case, the use of DMSO induces this effect on the CMC change at 37 °C. In systems where both PBS and DMSO are present, the electrolyte's effect of decreasing the CMC counteracts the effect of DMSO, which increases the CMC. Consequently, the CMC in these systems is lower than in the water-DMSO solution.

### 3.3 Lipopeptide selection

**Fig. 2:**
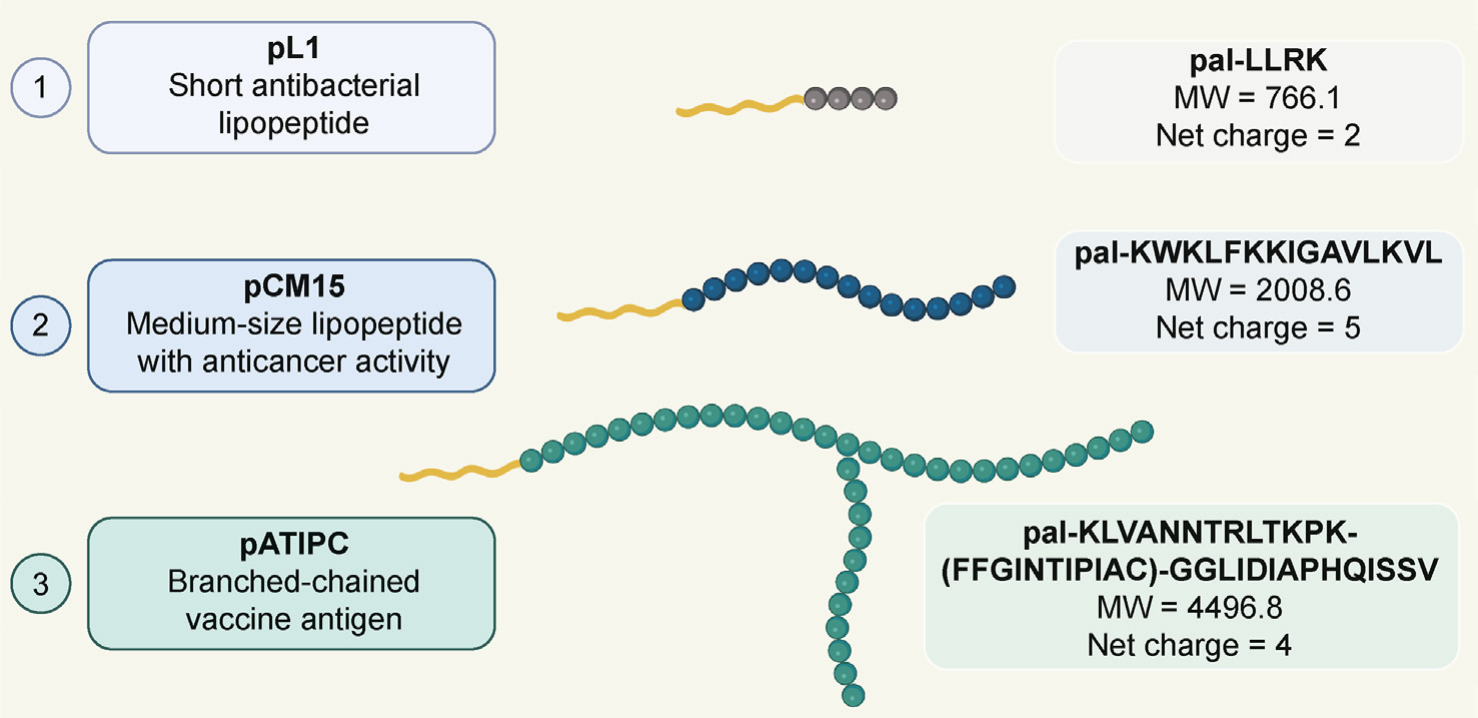
Lipopeptides with their characteristics, used in this study. MW: molecular weight. Figure was created with BioRender.com.

Three different model lipopeptides (Fig. 2) were designed and synthesized for this study:

i) A short cationic tetrapeptide, representing the active site [53] of a classical antimicrobial peptide (AMP), named Buforin II [54], was used in its lipidated form. Specifically, the N-terminus of the Leu-Leu-Arg-Lys tetrapeptide was amidated with palmitic acid. The resulting lipopeptide, palmitoyl-LLRK (pL1), exhibited potent antibacterial activity not only against the sensitive *S. aureus* but also against multidrug-resistant (MRSA) strains.
ii) The hybrid CM15 peptide (KWKLFKKIGAVLKVL), containing residues 1–7 of Cecropin and residues 2–9 of Melittin, has been reported to display potent antimicrobial and anticancer activity by disrupting cell membranes and cell walls [55,56]. The coupling of fatty acids to the CM15 peptide displays significantly higher anticancer activity compared to the parent peptide. Specifically, the C12 (lauric acid) and C16 (palmitic acid) elongated CM15 lipopeptide to form spherical nanoparticles or nanofibers, inducing cancer necrosis or necroptosis [57]. This medium-sized palmitoylated-CM15 lipopeptide was our second choice as a model compound.
iii) Pattern recognition receptors play important roles in the immune response to pathogens and vaccines. They recognize typical molecules of bacteria, primarily the lipidic components of the cell wall, such as liposaccharides and lipopeptides [58,59]. Therefore, elongating peptide epitopes with fatty acids can trigger the immune response and add an adjuvant effect to the antigens. Such self-adjuvanted multi-epitope construct was designed and synthesized in our laboratory, which showed relevant vaccine efficacy in a mice challenge model of *Mycobacterium tuberculosis* infection [60]. The fluorescently labelled derivative of this pATIPC construct was used in this study to evaluate the internalization properties into professional antigen presenting cells (APC), because the cellular uptake of APCs is a crucial step in the immune response to a certain vaccine.

Fatty acid elongation is a clinically validated modification technique that may significantly improve the pharmacological potency of biologically active peptides. The length of the fatty acid, the chemical linkage, and the site of lipidation all have crucial impact on the overall effect and biodistribution. Typically, increasing the length of the fatty acid chain results in increased cellular uptake. Compared to unmodified peptides, lipidation can improve the half-life mainly by enabling serum albumin binding. This can dramatically extend the inherently short half-life of peptides, to allow weekly or even longer dosing frequency. Besides, due to the enhanced cell membrane permeability and increased uptake, intracellular targets have become more reachable. Nevertheless, fatty acid elongation has opened new possibilities for different routes of administrations for peptide-based drug candidates (Table S1).

### 3.4 Characterization of the lipopeptide-loaded poloxamer micelles

Section S3 in the Supplementary data contains the detailed characterization of the mixed micelles (Fig. S7A). First, calibration curves were plotted for each lipopeptide in a medium in which the compounds were perfectly soluble, namely in eluent B (0.1% TFA in acetonitrile:water (4:1, *V/V*) solution) (Figs. S7B−D). Next, poloxamer-lipopeptide constructs were prepared as follows: the lipopeptide samples were pre-dissolved in DMSO and then mixed with the poloxamer/PBS solutions, left shaking overnight. In the next day, the concentration was adjusted by adding PBS to the solutions. The composition of the samples was as follows: poloxamer 5 g/L and peptides (pL1 200 μM, pCM15 100 μM, and pATIPC 10 μM). Then, lipopeptide/poloxamer constructs were centrifuged using a 0.2 μm nylon filter, diluted twice with eluent B, and injected to the HPLC column. From the area under the curve (AUC) data and the equation of the linear regression, the concentration of the lipopeptide, which is in a form of <0.2 μm, was calculated for each sample (Figs. S7E−G). The highest encapsulation efficacy was measured for the shortest lipopeptide pL1, and all three poloxamers formed mixed micelles with the lipopeptides in more than 80%. It was assumed that the filtered part of the samples contains the lipopeptide aggregates with particle size of >200 nm. For the medium-sized pCM15 peptides, Plur123 formulation resulted in more than 75% encapsulation, while around 60% encapsulation was observed for pATIPC. Overall, the lowest encapsulation efficacy and micelle formation capacity were observed for the pATIPC lipopeptide, probably because its branched structure hinders the polymer and lipopeptide chains from being arranged together.

#### 3.4.1 Structure of the mixed micelles

Afterward, DLS measurements were performed on the samples at 37.00 ± 0.02 °C. Well-defined systems with low polydispersity and particle sizes ranging from 19 to 32 nm were detected. The smallest particles were formed in the case of Plur123, while the largest particles were observed in the case of Plur127. The size change followed the same trend for all three peptides. The particle size was slightly larger in the case of pCM15 peptide (Table 4).

**Table 4:** Average hydrodynamic diameter (*d*_H_) and polydispersity indices (PDI) of the pL1, pCM15, and pATIPC peptide-loaded poloxamer micelles at 37 °C. The sample preparation and/or storage method is also marked.

Poloxamer	State	Lipopeptide-poloxamer mixed micelles					
		pL1		PCM15		pATIPC	
		*d*_H_ ± SD (nm)	PDI ± SD	*d*_H_ (nm) ± SD	PDI ± SD	*d*_H_ (nm) ± SD	PDI ± SD
Plur104	Fresh	24.8 ± 2.6	0.40 ± 0.30	24.6 ± 0.6	0.32 ± 0.03	20.9 ± 0.1	0.04 ± 0.01
Plur123	Fresh	18.7 ± 0.3	0.07 ± 0.05	22.7 ± 0.2	0.25 ± 0.03	19.8 ± 0.2	0.15 ± 0.02
Plur127	Fresh	28.2 ± 1.4	0.14 ± 0.05	32.1 ± 0.9	0.27 ± 0.10	27.5 ± 0.3	0.17 ± 0.09
Plur123	Stored at RT for one week	21.9 ± 2.1	0.25 ± 0.19	26.9 ± 0.7	0.07 ± 0.03	30.3 ± 0.7	0.62 ± 0.03
	Lyophilized-resuspended	19.2 ± 0.3	0.07 ± 0.02	19.5 ± 0.2	0.07 ± 0.03	19.6 ± 0.2	0.08 ± 0.02
	Frozen-thawed	18.2 ± 0.7	0.06 ± 0.04	19.6 ± 0.2	0.04 ± 0.02	19.2 ± 0.3	0.05 ± 0.02

SD: standard deviation; RT: room temperature.

The stability and storage ability of the mixed micelles were also examined by analyzing the lipopeptide-loaded Plur123 micelles. The results showed a slight increase in particle size for pL1 and pCM15 after one week of storage as a solution, while for pATIPC, the increase was significant, reaching 10 nm. However, when the fresh solution was lyophilized and recovered by adding water, followed by filtration, there was no change in particle size. The same observation was made when the solution was frozen, thawed, and then filtered. Overall, the polydispersity indices point to very stable systems (Table 4).

**Fig. 3:**
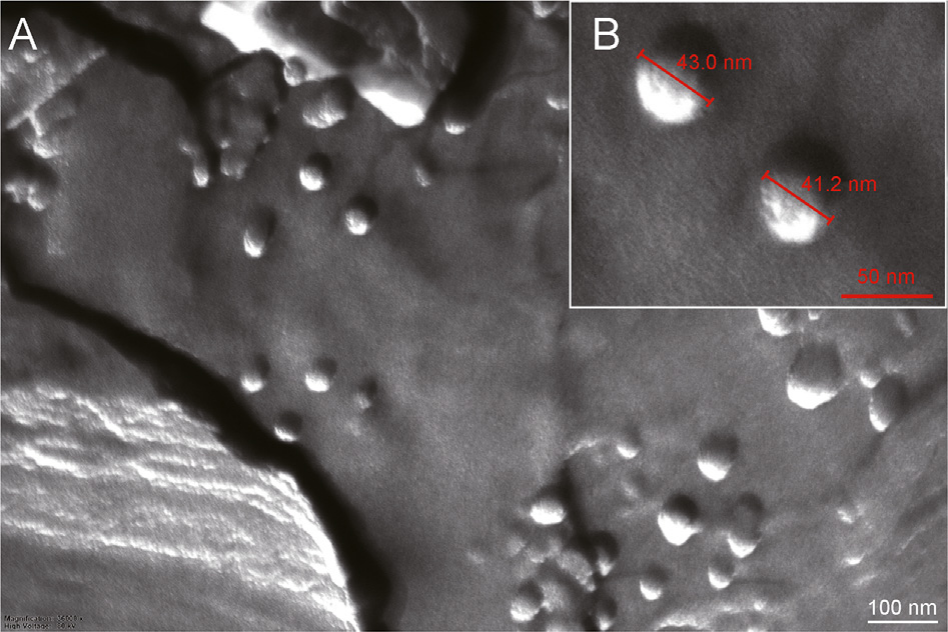
Freeze-fracture transmission electron microscopy (FF-TEM) images of pL1-Plur123 mixed micelles. Samples were prepared in distilled water, then glycerin was added, as a cryoprotective agent to avoid ice crystal formation. After ultrarapid freezing, fracturing was performed, and a replica was prepared by vaporized carbon and platinum/carbon evaporation. (A) Images of the replica were captured with a MORGAGNI 268D transmission electron microscope. (B) An enlarged image with the diameter marked.

The structure of the mixed micelles was also studied by FF-TEM. As a representative sample, the pL1-Plur123 system was processed. The freeze-fracturing technique is particularly useful for the study of biological or colloidal soft materials and contributes to the understanding of the morphology of polymer micelles in their relevant medium. Fig. 3 represents the FF-TEM images that show spherical particles with a flat surface. The size of the micelles is in agreement with the size obtained from the DLS measurements.

#### 3.4.2 IR spectroscopy

**Fig. 4:**
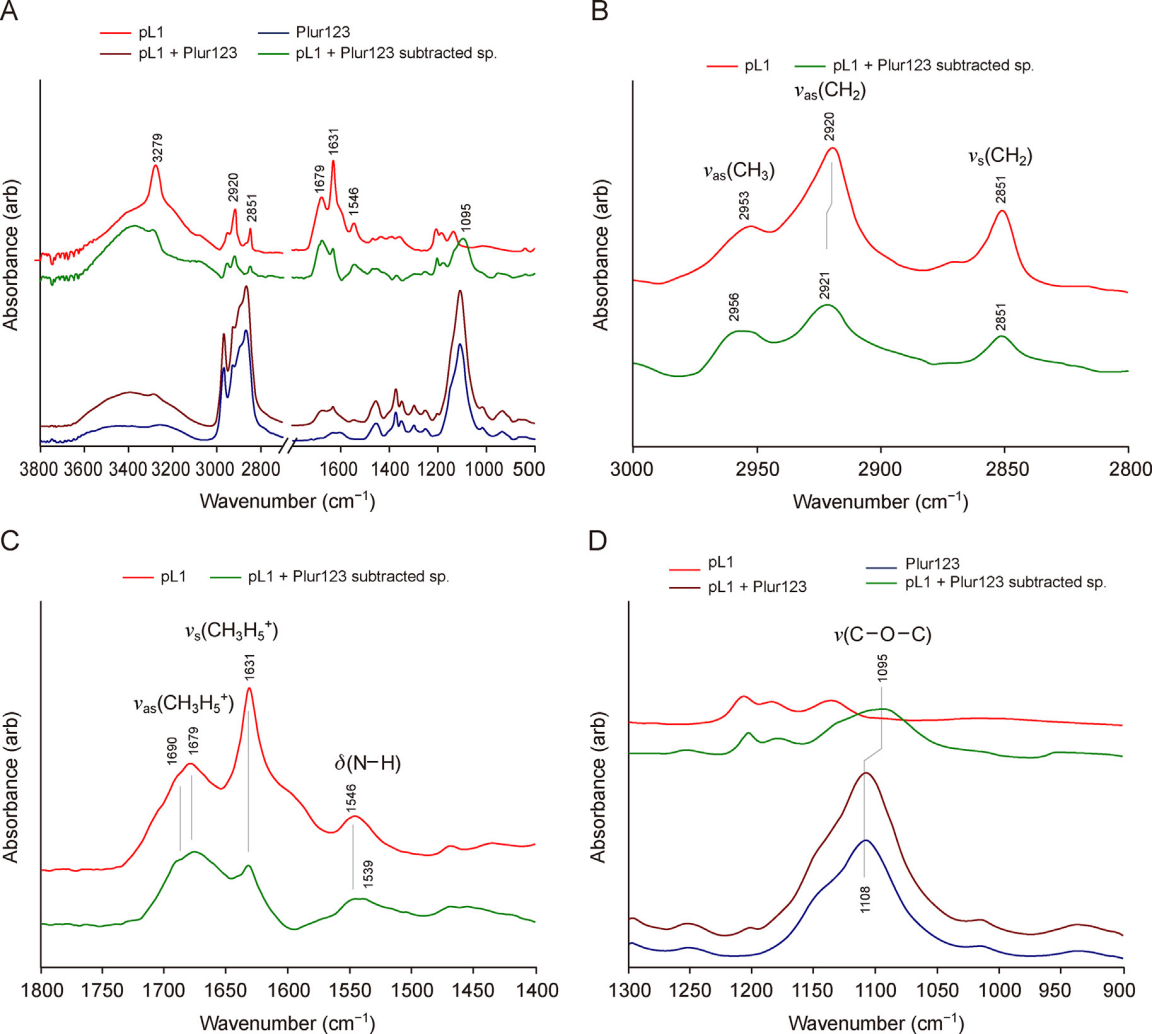
Structure characterization by infrared (IR) spectroscopy. (A) IR spectra of pure pL1 lipopeptide, Plur123, and pL1 + Plur123 assembly. To reveal spectral changes, the spectrum of Plur123 was subtracted from the spectrum of pL1 + Plur123 assembly, too (pL1 + Plur123 subtracted sp.). (B–D) Selected and enlarged parts of spectra, related to pL1 alkyl chains (B), peptide bonds (C), and C–O–C region of Plur123 (D), respectively. For better visualization, spectra are shifted vertically.

Compound pL1 and Plur123 were chosen as a model system to study the interaction between lipopeptides and poloxamers, and IR spectra were recorded using the ATR-IR technique (Fig. 4A). The spectrum of pL1 + Plur123 assembly resembles that of Plur123 one. However, to reveal nuanced spectral changes, the subtracted spectrum was created (the spectrum of pure Plur123 was subtracted from that of the pL1 + Plur123 assembly).

First, spectral change was observed regarding the palmityl alkyl chain of the pL1 lipopeptide (Fig. 4B). Upon interaction with Plur123, the antisymmetric (*v*_as_(CH_2_)) and symmetric (*v*_s_(CH_2_)) bands are diminished, broadened, and shifted towards higher wavenumber. Based on our previous experience with IR studies of lipid membranes composed from dipalmitoyl phosphatidyl choline [[61], [62], [63], [64], [65]], these changes are related to a decrease in alkyl chain packing and ordering.

Regarding the 1800–1400 cm^−1^ wavenumber region (Fig. 4C), characteristic bands of peptides are present. Contrary to proteins, in the case of short peptides, IR bands of acid side chains are dominating [[66], [67], [68]]. Indeed, the sharp, well-defined bands at 1679 and 1631 cm^−1^ in the spectrum of pL1 belong to vibrations of the guanidine group of arginine, *v*_as_(CN_3_H_5_^+^) and *v*_s_(CN_3_H_5_^+^), respectively [68]. The shoulder at 1690 cm^−1^ might belong to C=O stretching (*v*(C=O)) of peptide bonds, while the band at 1546 cm^−1^ can be assigned to N–H deformation (δ(N–H)). We have to point out that the relative intensity of *v*_s_(CN_3_H_5_^+^) is decreased. It seems plausible that there is a weak interaction between the C–O–C groups of Plur123 and the –NH_3_^+^ of arginine guanidine groups. This is further affirmed by the wavenumber shift of C–O–C stretching band (from 1108 to 1095 cm^−1^), noticeable in subtracted spectrum (pL1 + Plur123 subtracted spectrum, Fig. 4D).

As a summary, it can be stated that a weak interaction exists between pL1 and Plur123, involving guanidine groups and C–O–C groups, respectively. This interaction does not affect the peptide backbone/sidechain structure but cause a decrease in alkyl chain packing/order of pL1.

### 3.5 Biological evaluation

**Fig. 5:**
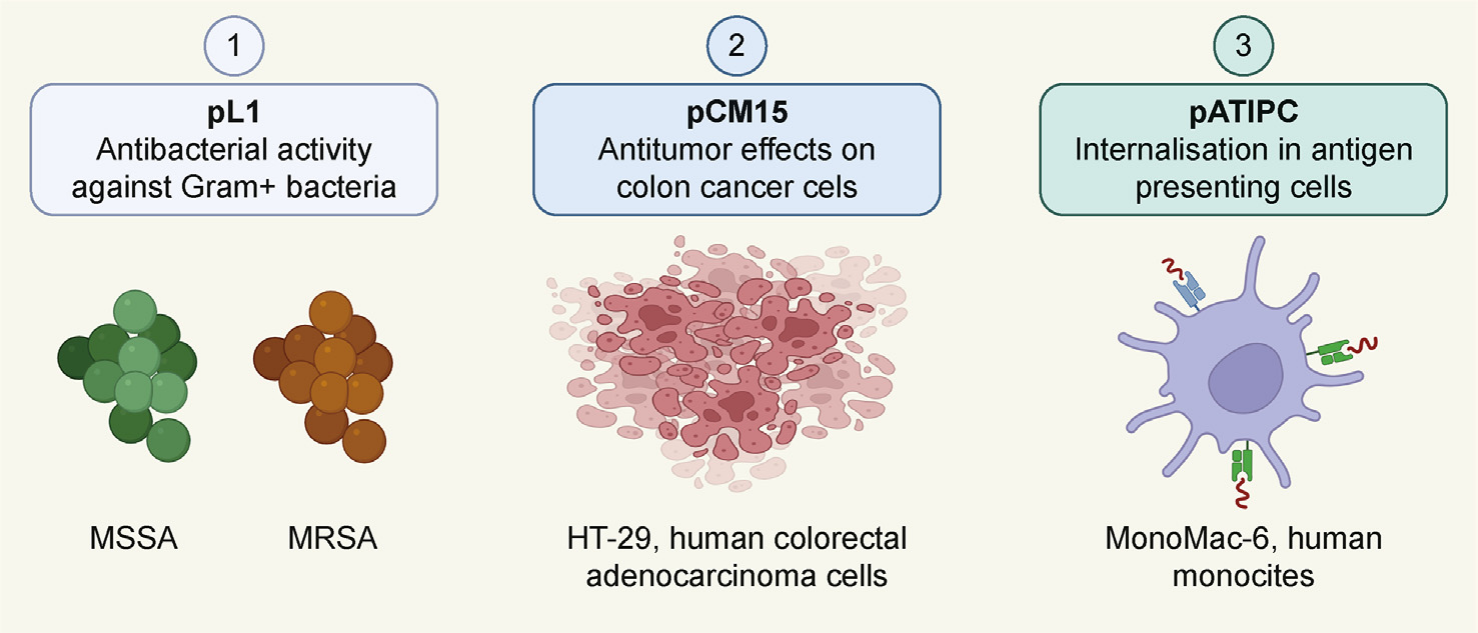
Schematic representation of the biological assays for the three different model peptides. MSSA: methicillin-susceptible *Staphylococcus aureus* (*S. aureus*); MRSA: methicillin-resistant *S*. *aureus*. Figure was created with BioRender.com.

The effect of the poloxamer dressing on the three different model lipopeptides was assessed in three distinct biological assays to determine their impact (Fig. 5).

#### 3.5.1 Antibacterial activity of pL1 peptide and its poloxamer derivatives

**Fig. 6:**
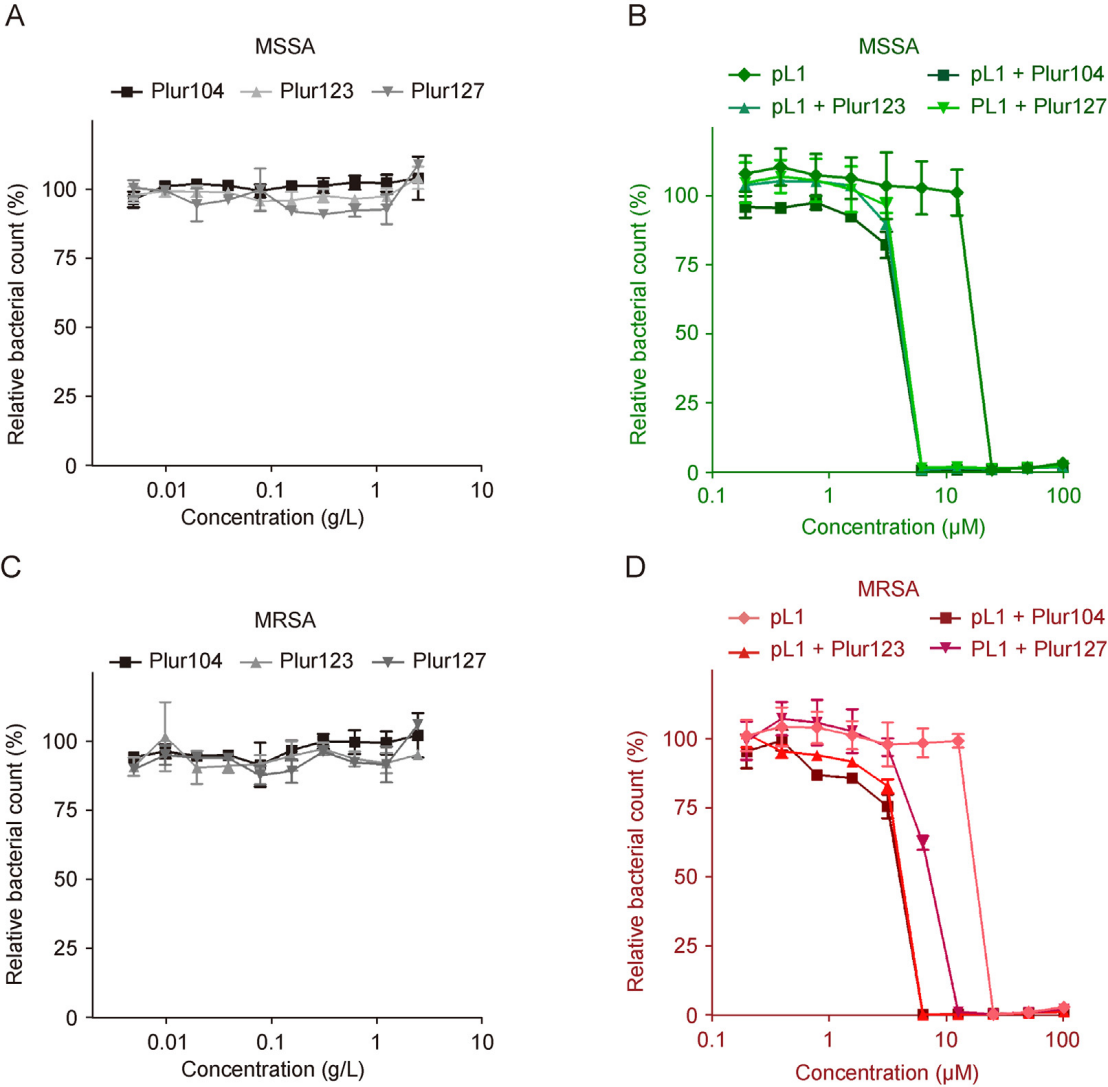
Antibacterial activity of the poloxamer polymers and the pL1-poloxamer constructs. (A, B) Methicillin-sensitive *Staphylococcus aureus* (*S. aureus*) (MSSA) strain (ATCC 29213) was used to test the poloxamers (A) and the mixed micelles (B) using standard broth-dilution method. (C, D) Methicillin-resistant *S. aureus* (MRSA) strain (ATCC 33591) was also tested for the poloxamers (C) and the lipopeptide constructs (D). Optical density (OD) was measured with a plate reader and means ± standard deviation (SD) were presented (*n =* 2).

Buforin I, which can be considered as the parent peptide of pL1 (sequence AGRGKQGGKVRAKAKTRSSRAGLQFPVGRVHRLLRKGNY), exhibited activity against *S. aureus* at 10 μg/mL, with an MIC of 14 μg/mL observed for the MRSA strain [69]. For the shorter Buforin II peptide (sequence: TRSSRAGLQFPVGRVHRLLRK), the published MIC values on *S. aureus* were between 4 and 8 μg/mL [53,70]. These MIC values correspond to a range of 1.6–3.3 μM. Lipopeptide pL1 exhibited antibacterial activity at slightly higher concentrations, ranging between 12.5 and 5 μM. However, since this peptide contains only four amino acids from the original Buforin sequence, it can be considered as a promising result. The poloxamer formulation significantly enhanced the antibacterial activity of pL1, reducing the MIC values to 3.1–6.3 μM (Fig. 6). Both the sensitive strain and the multiresistant *S. aureus* bacteria were effectively eradicated by pL1 and the poloxamer-pL1 constructs.

#### 3.5.2 Antitumor activity of pCM15 and its poloxamer derivatives on HT-29 cells

**Fig. 7:**
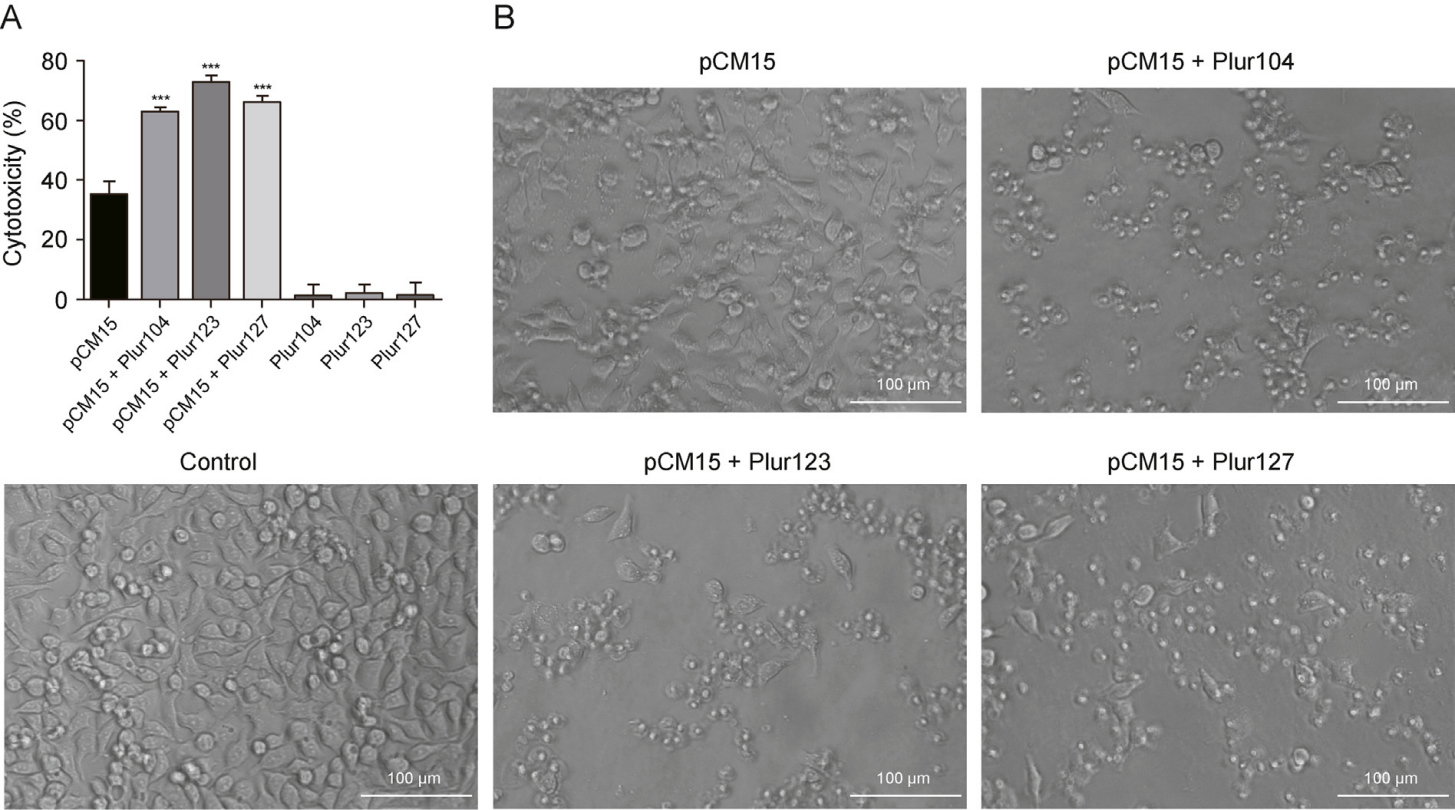
Antitumor activity of pCM15 lipopeptide and its poloxamer formulates. (A) The percentage of cytotoxicity caused by 5 μM pCM15 lipopeptide with or without poloxamer formulation (HT-29 colorectal cancer cells, 24 h of treatment), compared to medium-treated control cells. Poloxamer micelles, without pCM15 lipopeptides showed no cytotoxicity on HT-29 cells. (B) Changes in the cell morphology after treatment with the pCM15 lipopeptide construct. Images were captured after 24 h of treatment with an Olympus CX41 microscope. Statistical analysis was performed using one-way analysis of variance (ANOVA), followed by Tukey's post-hoc test. ^∗∗∗^*P* < 0.0001, compared with pCM15.

HT-29 is a human colorectal adenocarcinoma cell line characterized by epithelial morphology. These cells are commonly employed in the identification of novel chemotherapeutic agents against colorectal cancer and for drug development aimed at discovering more effective therapeutics [71,72]. Among other AMPs, CM15 was investigated on colorectal cancer cells, demonstrating promising antitumor activity [73]. Palmitoylated CM15 peptide was also evaluated against HT-29 cells, inducing approximately 30% cell death at a concentration of 5 μM after 1 h, and around 50% cell death after 24 h of treatment [57]. These data are consistent with our findings, as a cytotoxicity of 35.0% ± 4.8% was observed when cells were treated with 5 μM pCM15 lipopeptide for 24 h (Fig. 7). When poloxamer micelles were formed with the pCM15 lipopeptide, significantly higher antitumor activity was observed, namely 62.7% ± 1.8%, 71.2% ± 5.1%, and 67.2% ± 0.3% for pCM15 + Plur104, pCM15 + Plur123, and pCM15 + Plur127, respectively. Note that the pCM15 peptide content was the same for all three poloxamer-lipopeptide constructs. The improved antitumor activity is not caused by the cytotoxic effect of poloxamers, because poloxamer themselves do not directly induce death of HT-29 cells (Fig. 7).

#### 3.5.3 Internalization of pATIPC, a branched-chain multiepitope construct to model antigen-presenting cells

**Fig. 8:**
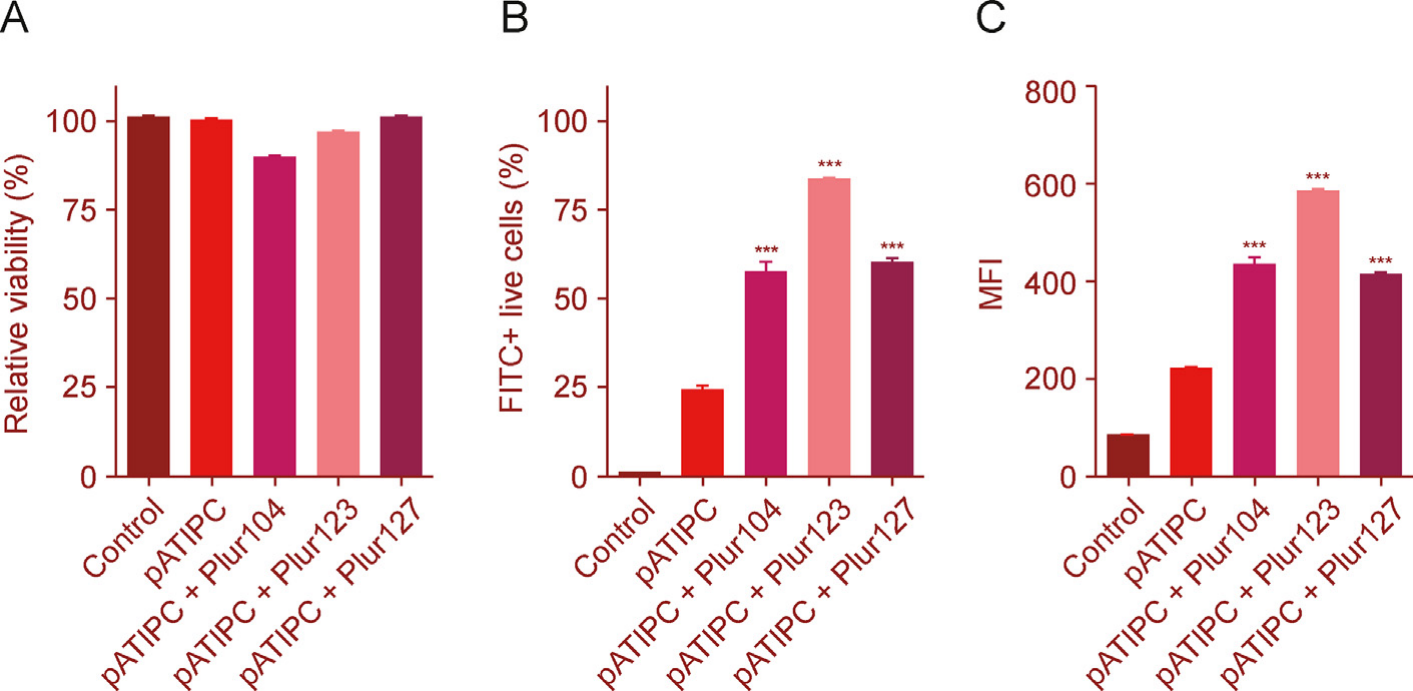
Cellular uptake of 5(6)-carboxyfluorescein (Cf)-labelled pATIPC vaccine conjugate. MonoMac-6 human monocytes were treated with 2.5 μM pATIPC lipopeptide with or without poloxamers. After 2 h, cells were washed and trypsinated in order to remove the outbound peptides. (A) Relative viability, compared to the medium-treated control cells, was determined first. (B, C) The percentage of live cells, which were peptide positive (B), together with the intracellular fluorescent intensity (mean fluorescent intensity (MFI)) (C) was measured. Bars represent the means ± standard deviation (SD) (*n =* 3), while the statistical analysis was performed using one-way analysis of variance (ANOVA), followed by Tukey's post-hoc test, ^∗∗∗^*P* < 0.0001, compared with unformulated pATIPC. FITC: fluorescein isothiocyanate.

Uptake by professional APCs, such as dendritic cells and macrophages, is a key element of the immune response induced by vaccine antigens. Nanoparticulated antigens, along with adjuvants, are of great interest as safer and more potent synthetic alternatives to classical vaccines. The lipid tail in the pATIPC multiepitope conjugate acts as a self-adjuvant moiety and improves the overall protective efficacy, as described in our previous paper [60]. Significantly higher splenocyte proliferation and production of interferon (IFN)-γ, interleukin (IL)-2, and IL-10 cytokines were measured for the palmitoylated conjugate compared to the epitope peptide mixture. Moreover, in a mice model of tuberculosis, the vaccine efficacy study revealed a significant decrease in the bacterial number observed in the spleen and in the lung, following immunization with pATIPC [60]. In this recent study, the uptake of poloxamer-formulated pATIPC by MonoMac-6 human monocytes, which can be considered as model APC, was evaluated (Fig. 8). A marked increase (*P* < 0.0001) in the internalization rate of the poloxamer-pATIPC micelles was found compared to the unformulated pATIPC lipopeptide. The best results were obtained for pATIPC + Plur123, with 83% of the MonoMac-6 cells being peptide-positive after treatment with as low as 2.5 μM lipopeptide encapsulated in Plur123.

To study the applicability of antigen-loaded poloxamer micelles as vaccines, the antigen release was investigated using a dialysis membrane assay (Section S3 in the Supplementary data). Results showed that even after 4 h, more than 50% of the pATIPC antigen remained inside the micelles, and the size and polydispersity of the nanoparticles were similar to the initial system (Fig. S8).

#### 3.5.4 Haemolytic activity assessment

**Fig. 9:**
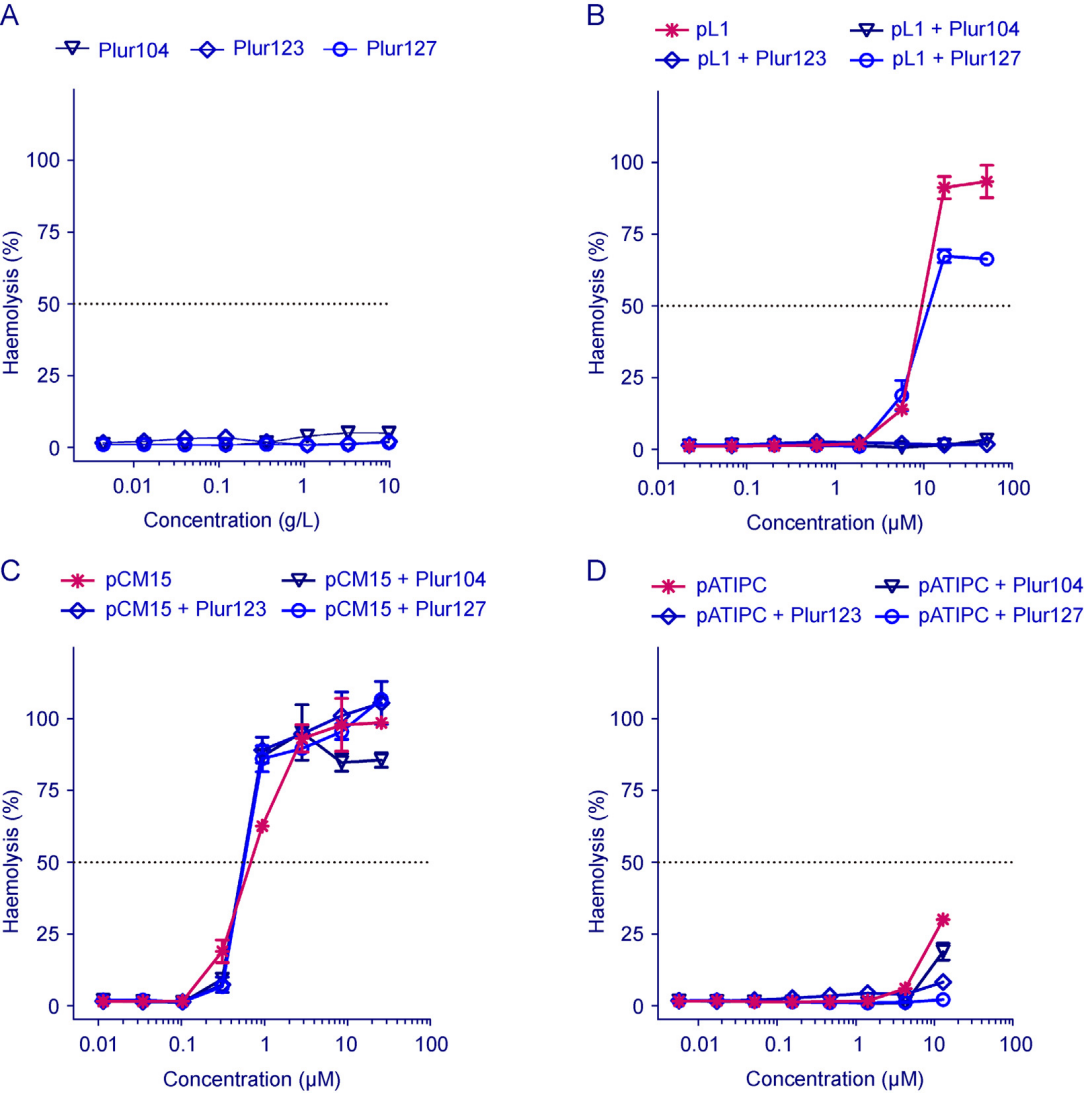
Haemolytic activity of poloxamers and lipopeptide-poloxamer constructs. Compounds were tested on human red blood cells (RBCs) (1% (*V/V*)) and the percentage of haemolysis, compared to medium-treated control wells, was plotted. Data were presented as mean ± standard deviation (SD) (*n =* 3).

In the following, the aim was to study how the selectivity of lipopeptides is affected by poloxamer dressing (Fig. 9). To explore this, a haemolysis assay was conducted, and the results were compared with the antibacterial and antitumor activity of the lipopeptides. In case of pL1, Plur127 dressing did not significantly alter the haemolytic activity. In contrast, Plur104 and Plur123 drastically altered the effect of the lipopeptide on human erythrocytes. Consequently, Plur104 and Plur123 significantly improved the selectivity of pL1 lipopeptide because these non-ionic surfactants enhanced the antibacterial activity while decreased the haemolytic activity. In case of pCM15, the haemolytic activity was not affected by the poloxamer formulation, and in the case of pATIPC, only slight changes were observed at the tested concentration range. Since this peptide was active at 2.5 μM, higher than 12.5 μM concentration was not tested in the haemolysis assay. Note that poloxamers themselves did not induce toxicity towards human erythrocytes up to 10 g/L concentration.

### 3.6 Overall characteristics of the lipopeptide-poloxamer mixed micelles

**Fig. 10:**
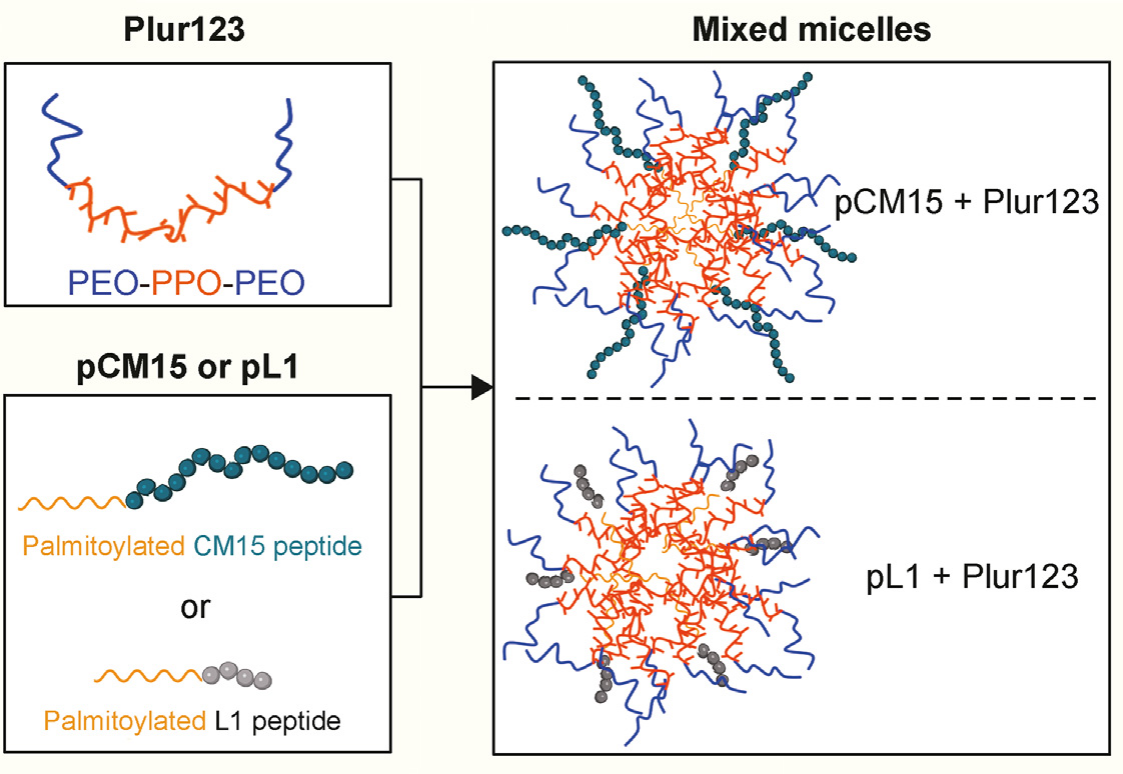
The schematic structures of pL1 and pCM15 lipopeptide containing mixed micelles. In the experimental samples, the peptide to poloxamer mass ratios are 1:25 and 1:33, respectively. In the case of pL1, the hydrophytic peptide chain is probably hindered by the of poly(ethylene oxide) (PEO) chains, while in the case of the longer pCM15, the C-terminus of the peptide is sticking out of the micelles. PPO: poly(propylene oxide). Figure was created with BioRender.com.

The most efficient entrapment and the most significant change due to poloxamer dressing were measured for the pL1 peptide. This phenomenon is believed to be attributed to the folding of the mixed micelles. In the case of pL1, the LLRK peptide chain is likely hidden between the hydrophilic PEO chains of poloxamer, whereas in the case of pCM15, the C-terminal of the peptide protrudes outside the micelle (Fig. 10). Note that the interaction of the PEO chains with the hydrophilic peptide part of pL1 was confirmed by the ATR-IR measurement. The branched chain arrangement in the pATIPC peptide probably prevents the formation of stable mixed micelles. This may cause formulation/solubilization being at its lowest level in this case. It is assumed that the fatty acid chain and the more hydrophobic side chain are both surrounded by the PPO chains of the poloxamers. In the case of the CM15 peptide, previous studies using molecular dynamics simulations [74] showed that the size of the extended disordered structure is around 5.1 nm, while the length of the more folded, helical structure is 2.5 nm. To estimate the length of the extended PEO chains and the PEO layer thickness, previous calculations and experimental data [75] were utilized. The radius of gyration (*R*_G_) can be calculated using the following equation:

*R*_G_ = *a*‧*N*^3/5^, where *a* is the size (0.35 nm) and *N* is the number of EO units in the PEO chain.

Considering this, the approximation for the PEO layer thickness in Plur123 is 4.2 nm. When comparing the length of the CM15 peptide and the thickness of the PEO layer, it's apparent that the C-terminal of the peptide can indeed be protruding, potentially influencing the properties of the mixed micelle. This is likely the reason why no decrease in activity of the CM15 peptide was observed in the haemolysis assay due to poloxamer dressing, whereas a significant decrease in haemolytic activity of the PL1 peptide was measured, where the peptide chain was masked.

## 4 Conclusions

This study outlines the design and synthesis of three lipopeptides with different properties: antibacterial and antitumor peptides, and a self-adjuvanting antigen. The objective was to nanoformulate these peptides into poloxamer micelles, characterize them, and investigate their biological properties. The long-term goal was to provide a method to render lipopeptides suitable for injection. Lipopeptides typically tend to form aggregates in the micrometer range, posing challenges for their intravenous injection. The pharmacopoeias impose strict regulations on particulate contamination of injectable preparations, because compound precipitation in injections can cause serious health problems such as the formation of blood clots or phlebitis. Poloxamer formulation presents a promising method to prevent precipitation and the formation of aggregates, ensuring that the resulted solutions are suitable for passage through an in-line filter. In case of vaccines, the storage and distribution in a freeze-dried form is very convenient. However, during reconstitution, it is essential to ensure that no aggregates or large particles are produced. Our results indicate that poloxamer-dressed lipopeptides are re-dissolvable after lyophilization and maintain a diameter similar to that of the original solution.

In order to determine the appropriate concentrations, information on the CMC of poloxamers was necessary, especially since various additives were introduced into our solutions. The literature contains numerous CMC data for the same copolymer, which may be due to different experimental conditions and varying homogeneity of the poloxamers. However, it should be noted that the CMC values of the applied poloxamers were not found in the same study or were inapplicable to our systems due to the presence of additives. It is worth mentioning that these CMC values were usually determined using different methods and not all required values could be collected. Therefore, it was decided to measure CMC values in aqueous, PBS, water-DMSO, and PBS-DMSO media at 25 and 37 °C using the pyrene fluorescent dye method. The obtained results are not only relevant for the present work but also for the systematic determination of CMC values, as three different hydrophobicity poloxamers were investigated under different additives and temperature conditions.

During the biological assessments, the properties of lipopeptide and lipopeptide-poloxamer systems were compared. The results showed that the cytotoxicity against tumor cells in the presence of poloxamers was approximately twice as high as that of the unformulated pCM15 lipopeptide. For the self-adjuvanted vaccine antigen, the rate of cellular uptake by antigen presenting model cells increased several-fold in the presence of poloxamers. Our most promising results were obtained with the ultrashort antimicrobial lipopeptide pL1. Its selectivity was significantly improved by the poloxamers, increasing its antibacterial activity while reducing its undesirable haemolytic activity. Among the poloxamers, the highest biological activity was observed when Plur123 was used.

Overall, it is concluded that poloxamer formulation is effective in optimizing the effect of lipopeptides, but the final properties of the mixed micelles are highly dependent on the length and structure of the peptide chain. It is believed that the optimal peptide chain length, that can still be hidden in the PEO chains of Plur123, is around 10–12 amino acids.

## CRediT authorship contribution statement

**Ágnes Ábrahám:** Investigation, Writing – original draft. **Gergő Gyulai:** Investigation, Methodology. **Judith Mihály:** Investigation, Methodology, Writing – original draft, Funding acquisition. **Andrea Horváth:** Investigation, Methodology, Writing – original draft. **Orsolya Dobay:** Investigation, Methodology, Writing – original draft. **Zoltán Varga:** Investigation, Methodology, Writing – review & editing, Funding acquisition. **Éva Kiss:** Supervision, Visualization, Writing – review & editing. **Kata Horváti:** Conceptualization, Investigation, Supervision, Visualization, Writing – review & editing.

## Declaration of competing interest

The authors declare that there are no conflicts of interest.
