# Supplementary material for: Optimizing lipopeptide bioactivity: The impact of non-ionic surfactant dressing
Source: J Pharm Anal. 2024 Jun 8;14(12):101020. doi: 10.1016/j.jpha.2024.101020 (PMC11774939; doi:10.1016/j.jpha.2024.101020)
Supplement: Multimedia component 1 [file mmc1.docx]

**Table S1.** Lipopeptide-based drugs, with their characteristics, disease target, and the year of approval [8,9].

| Generic name | Trade Name | Disease target | Administration | Approval Year | Half-life (h) | Plasma protein binding | Route of elimination |
| --- | --- | --- | --- | --- | --- | --- | --- |
| ***Antimicrobials*** | | | | | | | |
| Daptomycin (ss,c,C10) | *Cubicin, Dapzura* | Bacterial infections *caused by S. aureus,* skin structure infections | i.v. | 2003 | 7.5-9 | 90-94% | Urinary excretion |
|  |  |  |  | 2022 |  |  |  |
| Polymyxin B (ss,c,C9) | Polycin-B, Polytrim,, *Neo-Polycin, and others* | Bacterial infections | i.v. and topical use | 1951… | 6-11.5 | 79% to 92% | Mainly urinary excretion |
| Colistin  (ss,c,C8) | Cortisporin-TC, Xylistin, Coly-Mycin M, Colobreathe, and others | Bacterial infections, caused by Gram- bacilli | i.v. and topical use | 1968… | 5 | 50% | Mainly urinary excretion |
| Colistimethate (ss,c,C9) | *Colobreathe, Coly Mycin M, Xylistin* | Bacterial infections, caused by Gram- bacilli | i.v. | 2012 | 2-3 | nd. | Mainly urinary excretion |
| Dalbavancin (ss,c,C12) | *Dalvance* | Bacterial infections, caused by Gram+ bacilli | i.v. | 2014 | 346 | 93% | Mainly through fecal excretion |
| Caspofungin (ss,c,C16) | *Cancidas* | Esophageal candidiasis and invasive aspergillosis | i.v. | 2001 | 9-11 | 97% | Urinary and fecal excretion |
| Rezafungin (ss,c,C5) | *Rezzayo* | Candidemia and invasive candidiasis | i.v. | 2023 | >130 | 87-99% | Mainly through fecal excretion |
| Micafungin (ss,c,C5) | *Mycamine* | treatment and prophylaxis of Candida infections | i.v. | 2005 | 14-17 | >99% | Mainly through fecal excretion |
| ***Long-acting insulins*** | | | | | | | |
| Insulin detemir (s,c,C14) | *Levemir* | Type 1 and type 2 diabetes | s.c. | 2005 | 5-7 | 98% | Mainly urinary excretion |
| Insulin degludec (s,c,C16) | Ryzodeg, Tresiba, Xultophy | Type 1 and type 2 diabetes | s.c. | 2015 | 25 | >99% | Mainly urinary excretion |
| ***GLP-1 receptor agonists*** | | | | | | | |
| Liraglutide (s,l,C16) | Saxenda, Victoza, Xultophy | Type 2 diabetes | s.c. | 2010 | 13 | 98% | Urinary and fecal excretion |
|  |  | Obesity | s.c. | 2014 |  |  |  |
| Semaglutide (s,l,C18) | *Ozempic*  *Rybelsus*  *Wegovy* | Type 2 diabetes | s.c. | 2017 | 168 | >99% | Urinary and fecal excretion |
|  |  |  | Oral | 2019 |  |  |  |
|  |  | Obesity | s.c. | 2021 |  |  |  |
| Tirzepatide (s,l,C20) | *Mounjaro, Zepbound* | Type 2 diabetes | s.c. | 2022 | 120 | 99% | Urinary and fecal excretion |
|  |  | Obesity | s.c. | 2023 |  |  |  |
| ***Other*** | | | | | | | |
| Tesamorelin (s,l,C6) | *Egrifta SV* | Lipodystrophy in HIV patients | i.v. | 2010 | 0.4-0.6 | nd. | Mainly urinary excretion |

Abbreviations below the generic names: synthetic(s), semi-synthetic(ss), linear (l), cyclic (c), length of fatty acids (C5-C20).

nd: no data available, GLP-1: Glucagon-like peptide-1, s.c.: subcutaneous injection, i.v. intravenous injection.

**Table S2**. Critical micelle concentration (CMC) range of the poloxamers at 37°C based on literature data [13,23–29].

| Poloxamer | CMC at 37 °C | |
| --- | --- | --- |
|  | (µM) | (g/L) |
| Plur64 | 480 | 1.39 |
| Plur68 | 480 | 4.03 |
| Plur84 | 71-357 | 0.3-1.5 |
| Plur103 | 4-6.1 | 0.02-0.03 |
| Plur104 | 3.4-13 | 0.02-0.08 |
| Plur105 | 6.2-15 | 0.040-0.097 |
| Plur123 | 2-4.4 | 0.010-0.022 |
| Plur127 | 2.8-19 | 0.034-0.23 |
