# Supplementary material for: Optimizing lipopeptide bioactivity: The impact of non-ionic surfactant dressing
Source: J Pharm Anal. 2024 Jun 8;14(12):101020. doi: 10.1016/j.jpha.2024.101020 (PMC11774939; doi:10.1016/j.jpha.2024.101020)
Supplement: Multimedia component 2 [file mmc2.docx]

**Supplementary data**

**Optimizing lipopeptide bioactivity: The impact of non-ionic surfactant dressing**

**Table of Contents:**

**S1. Lipopeptides**

*S1.1. Detailed Synthesis of the Lipopeptides*

*S1.2. High-Resolution Mass Spectrometric Analysis of the Lipopeptides*

**S2. Poloxamers**

*S2.1. Critical Micelle Concentration (CMC) of the Selected Poloxamer*

*S2.2. Cytotoxicity of the Selected Poloxamers*

**S3. Peptide-Loaded Poloxamer Micelles**

*S3.1. Characterization of the Peptide-Loaded Poloxamer Micelles*

*S3.2. Antigen Release from the pATIPC-Plur123 Mixed Micelles*

**Table S1.** Lipopeptide-based drugs, with their characteristics, disease target, and the year of approval.

**Table S2**. Critical micelle concentration (CMC) range of the poloxamers at 37°C based on literature data.

**S1. Lipopeptides**

*S1.1. Detailed Synthesis of the Lipopeptides*

The instrument used for the preparation of CM15 and ATIPC is a commercially available flow peptide equipment (HPPS-4000, METALON Ltd., Budapest Hungary) consisting of a conventional Jasco LC-4000 series HPLC system, except for the PU-4180 HPLC pump, modified with an additional valve to allow recirculation and regulation of solvent flow (e.g. cleavage mixture). The ChromNAV2 software ensures a fully automated process. The autosampler injects the reagent solutions from a 1.5 mL sample vial placed in the sample rack. The PEEK chromatography column was used as the fixed bed reactor tube for the resin (130-150 mg of TentaGel S RinkAmide (capacity = 0.23 mmol/g)) and DMF was used as the eluent. An NMP stock solution was prepared for each standard amino acid and placed in a vial with the appropriate amount of OxymaPure. The amount of the injected amino acids and OxymaPure were 3 equivalents, calculated to the resin capacity. The autoinjector mixed the DIC (6 equivalent) and injected the activated solution into the system. For Fmoc cleavage, 0.6 ml of 30 *V/V*% piperidine/DMF solution was used. During the synthesis, the pressure was varied between 60 and 80 bar using a back pressure regulator at 80 °C. Initially, the flow rate was 0.3 mL/min (during the coupling) which was changed gradually from 0.3 to 1.0 mL/min. It remained until the end of the cycle (during the Fmoc-cleavage) and then returned to its initial value of 0.3 mL/min, requiring a cycle time 12 min in total. After synthesis, the resin was washed with DCM.

Palmitic acid was coupled to the *N*-terminus of each peptide using DIC/HOBt coupling method. Peptides were cleaved from the resin with TFA/H_2_O/TIS (94 : 3 : 3 *V/V*) mixture (2 h, RT). After filtration, compounds were precipitated in cold diethyl ether, centrifuged (4000 rpm, 5 min) and freeze-dried from water. The branched chain of the pATIPC conjugate was prepared using thiol/maleimide coupling method, as detailed previously.

*S1.2. High-Resolution Mass Spectrometric (HRMS) Analysis of the Lipopeptides*


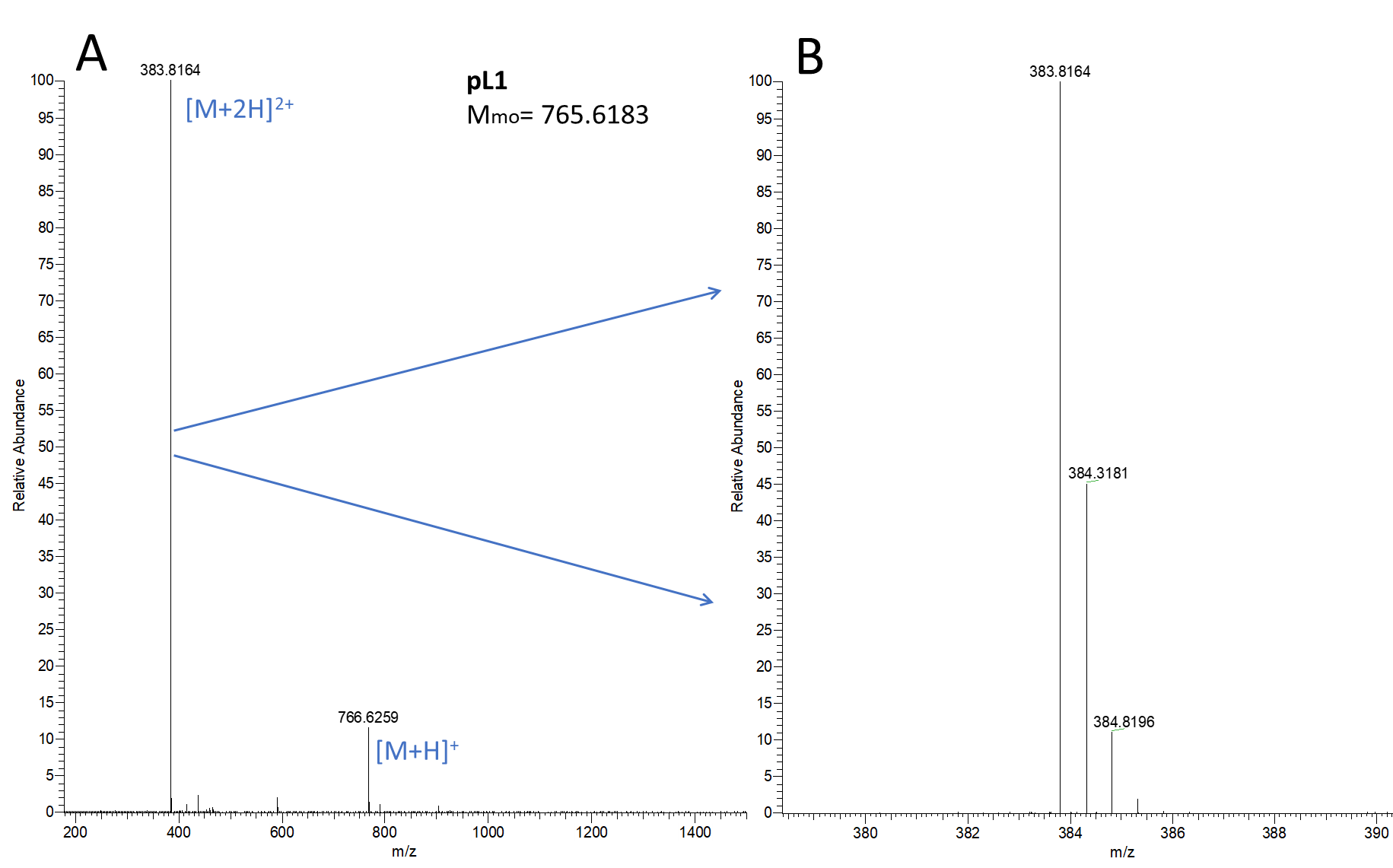


**Fig. S1**. HRMS spectrum of pL1 lipopeptide. Full *m/z* range (**A**) and the magnification of the peak corresponding to the [M+2H]^2+^ ion (**B**).


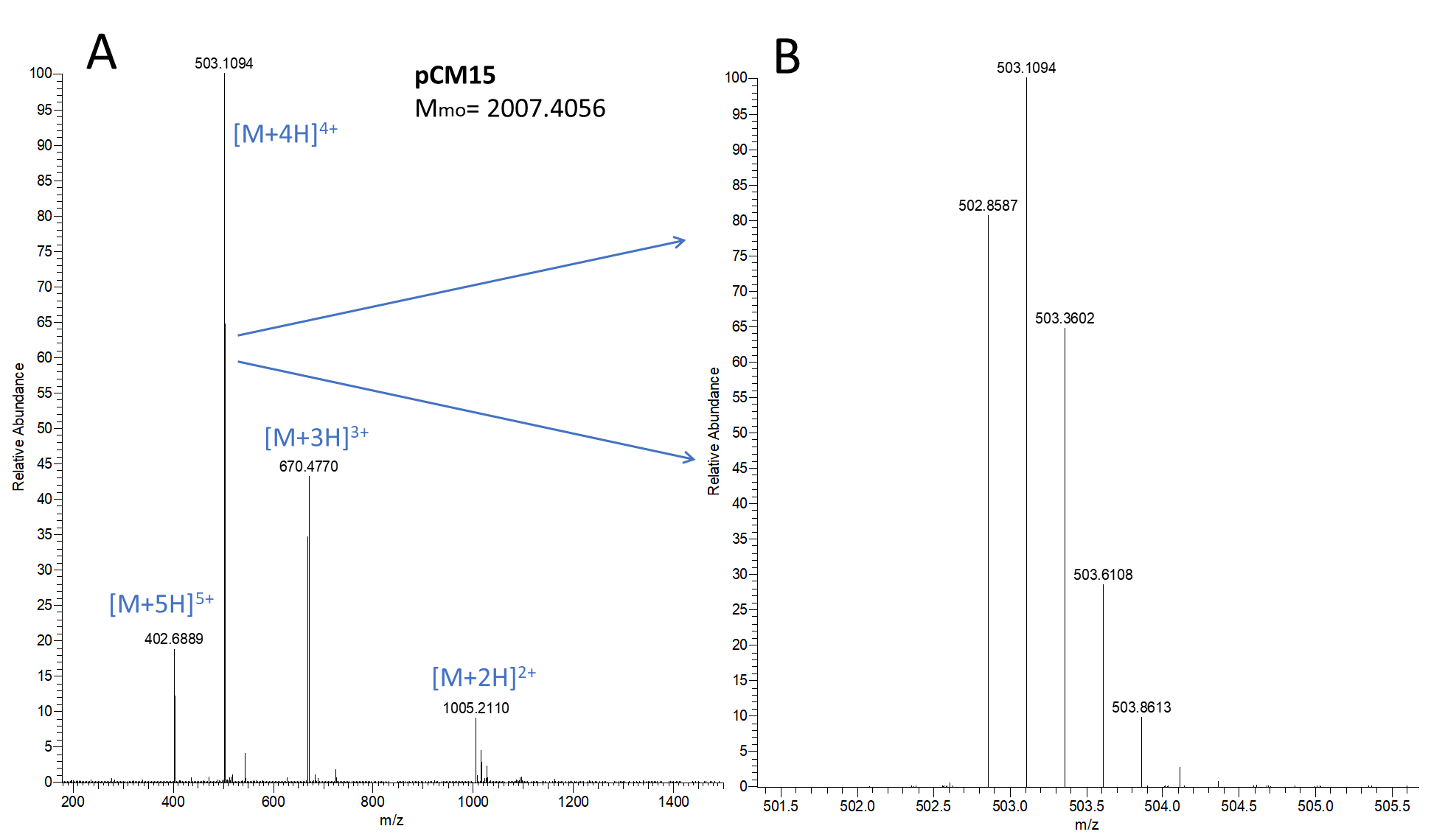


**Fig. S2**. HRMS spectrum of pCM15 lipopeptide. Full *m/z* range (**A**) and the magnification of the peak corresponding to the [M+4H]^4+^ ion (**B**).


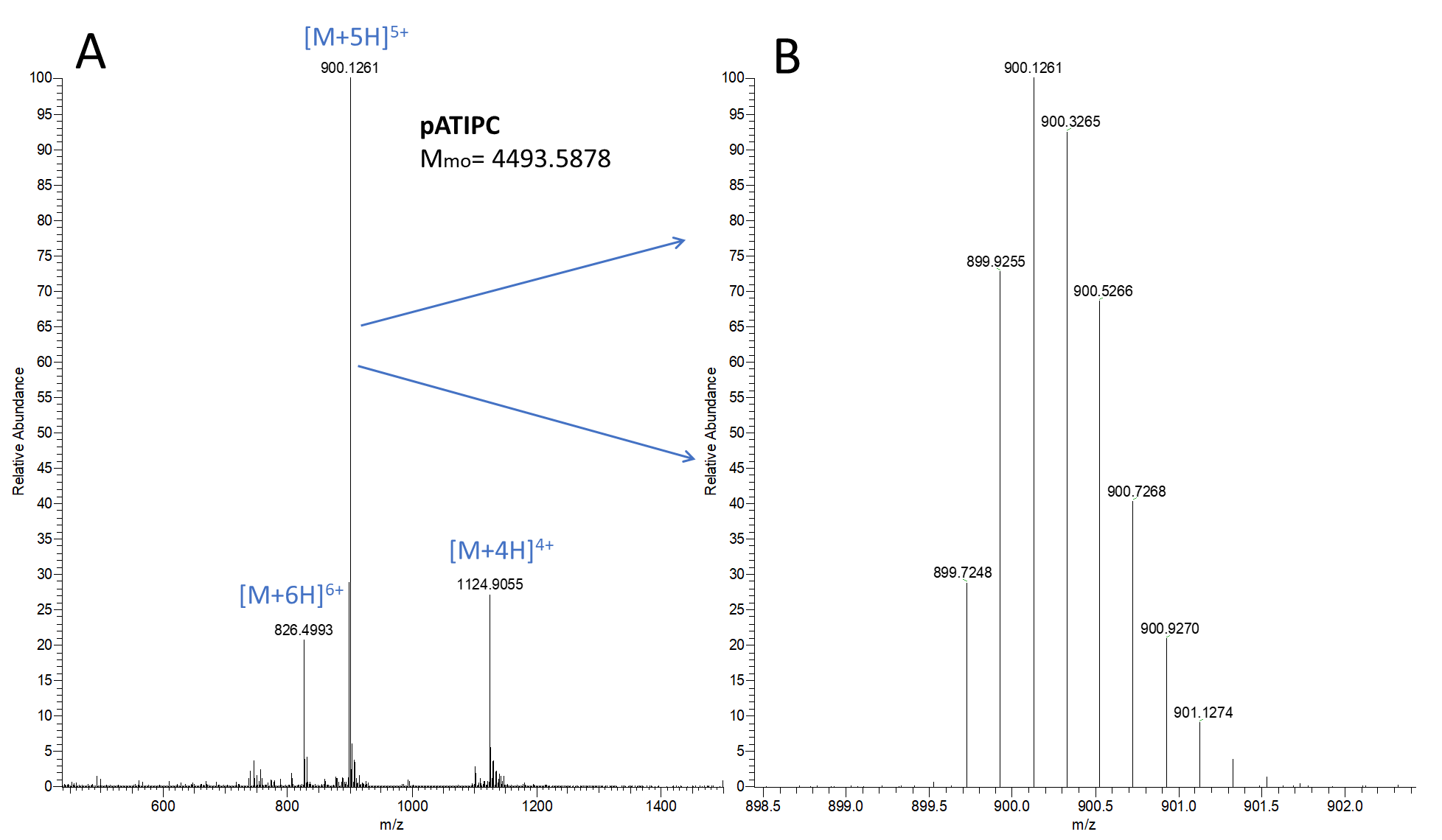


**Fig. S3**. HRMS spectrum of pATIPC lipopeptide. Full *m/z* range (**A**) and the magnification of the peak corresponding to the [M+5H]^5+^ ion (**B**).

**S2. Poloxamers**

*S2.1. Critical Micelle Concentration (CMC) of the Selected Poloxamer*

The critical micelle concentrations of the poloxamers in various media were determined at 25 and 37°C applying the pyrene fluorescent probe method.

Based on the measurement data, the I_1_/I_3_ vs log *c* graph can be constructed (**Fig. S1**). With increasing concentration of the poloxamers, at a specific point a significant decrease in the I_1_/I_3_ values can be observed. This decline indicates the formation of micelles and the emergence of hydrophobic domains. Towards the end of this transition concentration range, the solubilization of pyrene reaches saturation, as evidenced by a consistently low value of I_1_/I_3_.





**Fig. S4.** Pyrene I_1_/I_3_ values as a function of the natural logarithm of Plur104 concentration at 37 °C in water (black), in PBS (red), in water with 5% DMSO content (green) and in PBS with 5% DMSO content (blue) media.

The data points obtained from the measurements were fitted using a decreasing Boltzmann-type sigmoid described by Equation (1).

$y=\frac{A_{1}-A_{2}}{1+e^{(x-x_{0})/\Delta x}}+A_{2}$ (1)

In this equation, the variable y represents the I_1_/I_3_ values, while x represents the total poloxamer concentration. A_1_ and A_2_ are the upper and lower limiting values, x_0_ is the center of the sigmoid and Δx is the width of the sigmoid. There has been a discussion in the literature regarding which point on the curve should be used to determine the CMC. Based on this, the CMC should be given as x_0_ if the ratio x_0_/Δx<is less than 10 and as (x_0_+2Δx) otherwise. Since the condition x_0_/Δx<10 was valid in all our cases, x_0_ was selected as the CMC.

*Section S2.2. Cytotoxicity of the Selected Poloxamers*


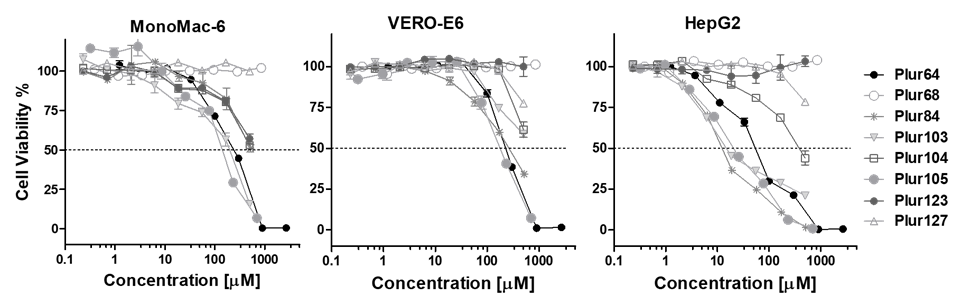


**Fig. S5.** Cell viability of MonoMac-6 human monocytes, VERO-E6 kidney cells and HepG2 human hepatoma cells, after the treatment with poloxamers for 24 hours. The cell viability was determined by AlmarBlue assay and compared to the medium-treated cells. Means of 4 data ± Sd are presented.


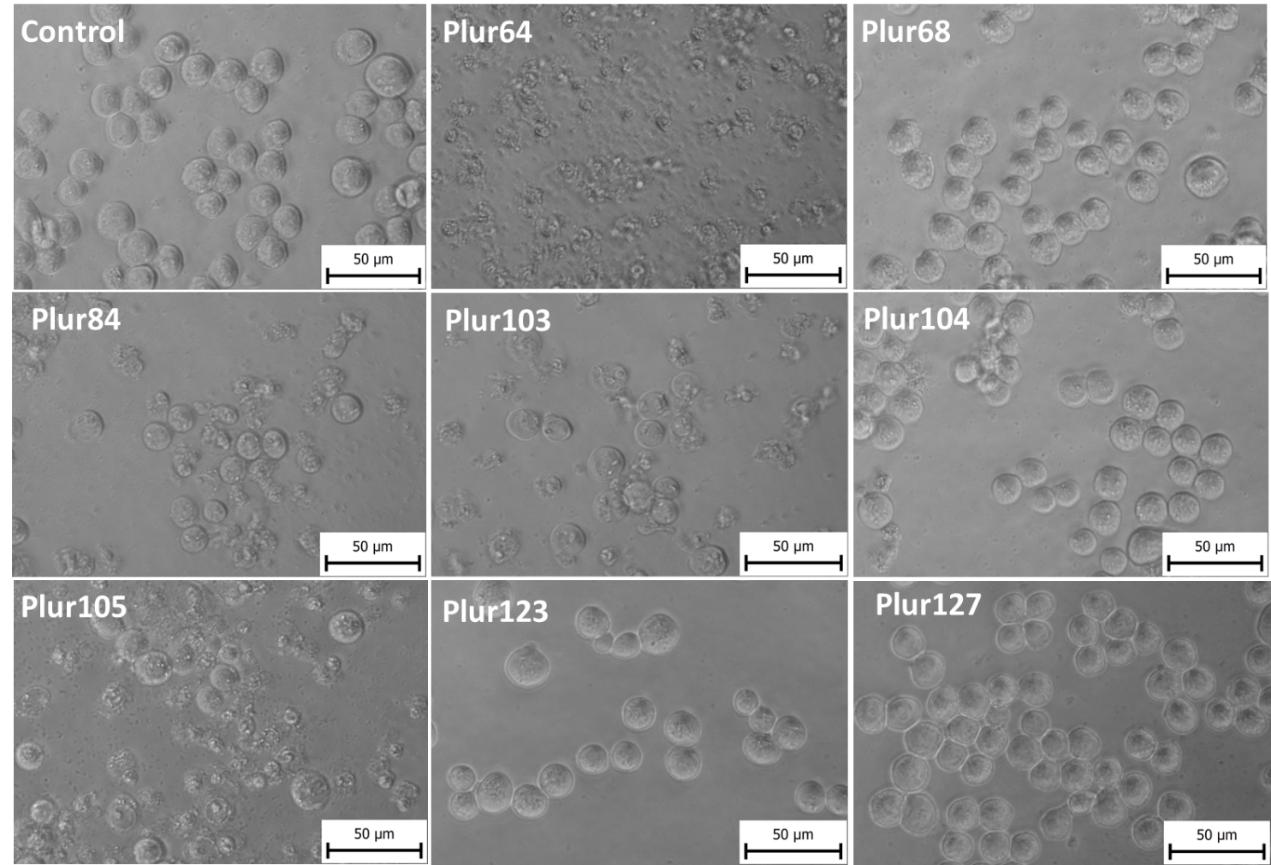


**Fig. S6.** Microscopic images of MonoMac-6 human monocytes, treated with different poloxamers (highest concentration, as indicated in **Table S2,** typically between 2 – 8 g/L). Images were captured after 24 hours of incubation using an Olympus CX41 microscope (objective 40X).

**S3. Peptide-Loaded Poloxamer Micelles**

*S3.1. Characterization of the Peptide-Loaded Poloxamer Micelles*


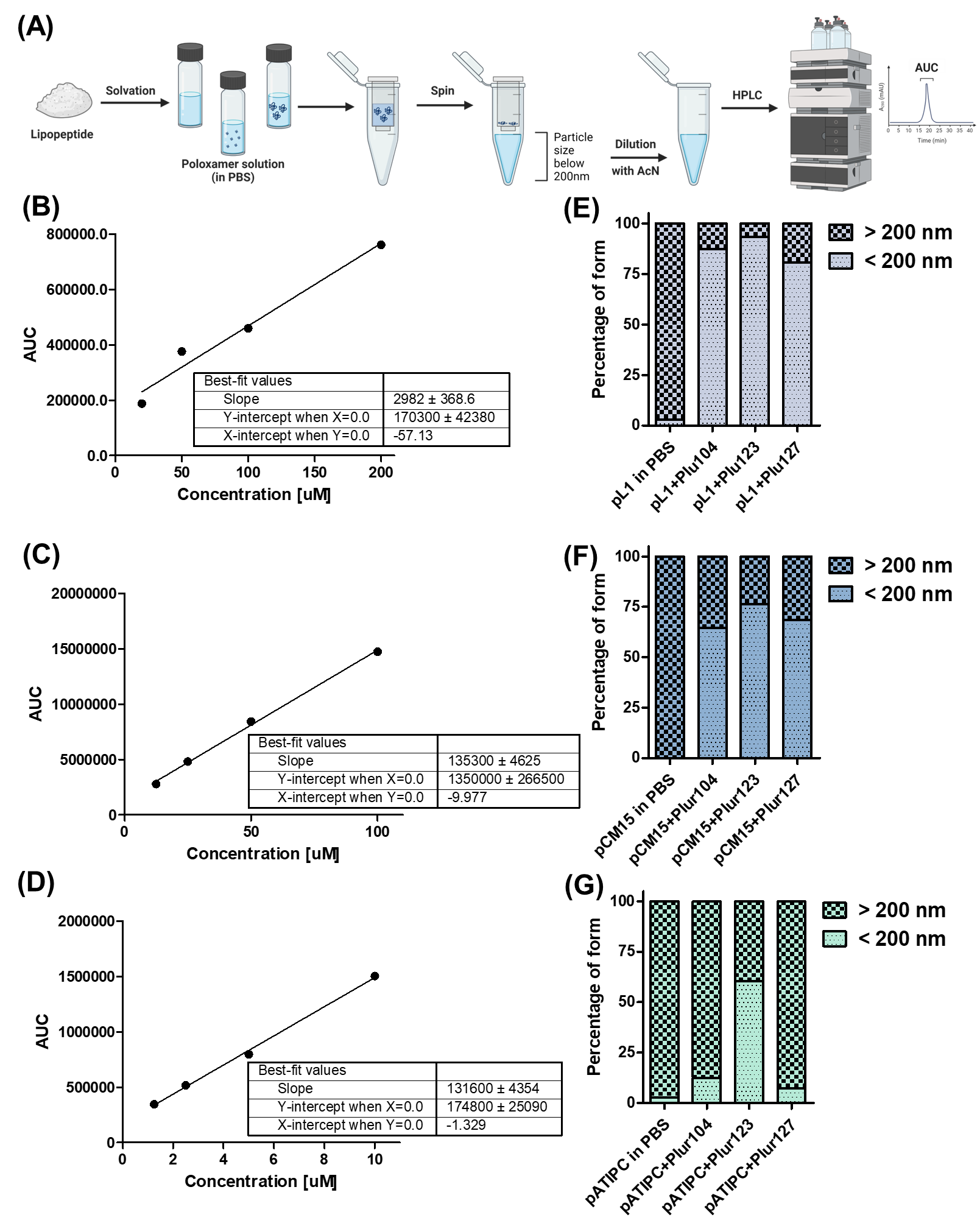


**Fig. S7.** **Formulation of Lipopeptides with Poloxamers.** Overview of the analytical characterization of the samples (A). Calibration curves for the fully solubilized lipopeptides with the linear regression equation (B-D). The goodness of fit (r^2^) was 0.9703 for pL1; 0.9977 for pCM15 and 0.9978 for pATIPC. Panels (**E-G)** show the percentage of lipopeptides in a form of below and above 200 nm particle size. One hundred µM pL1 peptide, dissolved in PBS or formulated with Poloxamers was plotted on panel (**E)**, while 50 µM pCM15 on panel (**F)** and 5 µM pATIPC on panel **(G).** Percentage of form was defined as the ratio of filtered samples (< 200 nm), compare to the perfectly solubilized lipopeptide (set as 100%). AUC: Area under the curve.

*S3.2.* *Antigen Release from the pATIPC-Plur123 Mixed Micelles*

Lipopeptide release was assessed by incubating the mixed micelles in PBS at 37^o^C for 6 hours, using the dialysis membrane method. Briefly, the pATIPC lipopeptide (80 µM) was dissolved in Plur123 (40 g/L, in PBS), mixed, then centrifuged using a 0.2 µm Nylon filter to remove the aggregated peptide particles. The formation of the mixed micelles was checked by DLS and the peptide content was measured by HPLC, as described above. Mixed micelle solution was then loaded into a Slide-A-Lyzer Mini Dialysis Device (10K MWCO) (Thermo Fisher Scientific, Waltham, MA, USA) and incubated at 37 ^o^C using PBS as release medium. At 0, 1, 4 and 6 h, the micelles within the dialysis membrane were taken for measurements of particle size and distribution by DLS. Subsequently, 75 µL sample was diluted with 75 µL eluent B (0.1% TFA in acetonitrile/water = 80/20 (*V/V*) and assayed by HPLC. Based on the AUC data, the pATIPC release percentage was calculated compared to the sample peptide content at time 0 h (**Fig. S8**).


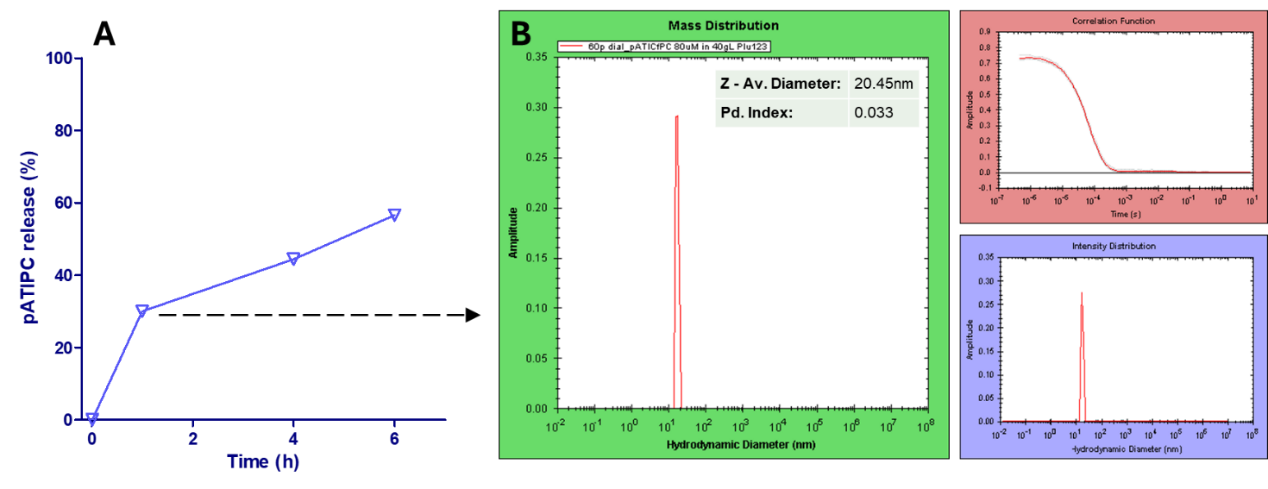


**Fig. S8**. Antigen release from the poloxamer-lipopeptide mixed micelles. The release assay was performed using the dialysis membrane method, by applying a Molecular weight cut-off (MWCO) bigger than the peptide, but smaller than the micelles (**A**). Hydrodynamic diameter of the nanoparticles and the corresponding PDI was measured by DLS (**B**).
